# Evidence for genetic contribution to the increased risk of type 2 diabetes in schizophrenia

**DOI:** 10.1038/s41398-018-0304-6

**Published:** 2018-11-23

**Authors:** Sophie Hackinger, Bram Prins, Vasiliki Mamakou, Eleni Zengini, Eirini Marouli, Luka Brčić, Ioannis Serafetinidis, Klea Lamnissou, Vassilis Kontaxakis, George Dedoussis, Fragiskos Gonidakis, Anastasia Thanopoulou, Nikolaos Tentolouris, Aspasia Tsezou, Eleftheria Zeggini

**Affiliations:** 1 Human Genetics, Wellcome Trust Sanger Institute, Hinxton, CB10 1HH UK; 2 Strangeways Research Laboratory, University of Cambridge, 2 Worts’ Causeway, Cambridge, CB1 8RN UK; 3 Medical School, National and Kapodistrian University of Athens, Athens, 11528 Greece; 4 5th Department, Dromokaiteio Psychiatric Hospital, Athens, 124 61 Greece; 5 Department of Oncology and Metabolism, University of Sheffield, Beech Hill Road, Sheffield, S10 2RX UK; 6 William Harvey Research Institute, Barts and The London School of Medicine and Dentistry, Queen Mary University of London, London, EC1M 6BQ UK; 7 University of Split, Livanjska ul. 5, Split, 21000 Croatia; 8 Department of Gastroenterology, Gennimatas General Hospital, Athens, 11527 Greece; 9 Department of Biology, National and Kapodistrian University of Athens, Athens, Panepistimioupolis, Ano Ilisia, Athens, 15771 Greece; 10 Early Psychosis Unit, 1st Department of Psychiatry, Eginition Hospital, Medical School, National and Kapodistrian University of Athens, Athens, 11527 Greece; 11 Department of Nutrition-Dietetics, Harokopio University, 17671 Athens, Greece; 12 1st Department of Psychiatry, Eginition Hospital, Medical School, National and Kapodistrian University of Athens, Athens, 11528 Greece; 13 Diabetes Centre, 2nd Department of Internal Medicine, Hippokration General Hospital, Medical School, National and Kapodistrian University of Athens, Athens, 11527 Greece; 14 1st Department of Propaedeutic and Internal Medicine, National and Kapodistrian University of Athens, Medical School, Laiko General Hospital, Athens, 11527 Greece; 15 Faculty of Medicine, Department of Biology, University of Thessaly, 41500 Larissa, Greece; 16 Institute of Translational Genomics, Helmholtz Zentrum München, German Research Center for Environmental Health, Neuherberg, Germany

## Abstract

The epidemiologic link between schizophrenia (SCZ) and type 2 diabetes (T2D) remains poorly understood. Here, we investigate the presence and extent of a shared genetic background between SCZ and T2D using genome-wide approaches. We performed a genome-wide association study (GWAS) and polygenic risk score analysis in a Greek sample collection (GOMAP) comprising three patient groups: SCZ only (*n* = 924), T2D only (*n* = 822), comorbid SCZ and T2D (*n* = 505); samples from two separate Greek cohorts were used as population-based controls (*n* = 1,125). We used genome-wide summary statistics from two large-scale GWAS of SCZ and T2D from the PGC and DIAGRAM consortia, respectively, to perform genetic overlap analyses, including a regional colocalisation test. We show for the first time that patients with comorbid SCZ and T2D have a higher genetic predisposition to both disorders compared to controls. We identify five genomic regions with evidence of colocalising SCZ and T2D signals, three of which contain known loci for both diseases. We also observe a significant excess of shared association signals between SCZ and T2D at nine out of ten investigated *p* value thresholds. Finally, we identify 29 genes associated with both T2D and SCZ, several of which have been implicated in biological processes relevant to these disorders. Together our results demonstrate that the observed comorbidity between SCZ and T2D is at least in part due to shared genetic mechanisms.

## Introduction

Schizophrenia (SCZ) patients are 1.5–2 times more likely to develop type 2 diabetes (T2D) compared to the general population^1^. Several explanations for this epidemiologic link have been proposed, including environmental factors, the use of antipsychotic medication, and/or shared genetic aetiology^1–4^. For example, patients with severe mental illness often lead a more sedentary life and are more likely to smoke compared to the general population^4^—both risk factors for T2D. Antipsychotic drugs, particularly second generation antipsychotics, are known to cause metabolic side effects and often lead to significant weight gain^5^. Several studies have found an association between psychotropic medication and T2D risk^6–8^, but it is still unclear to what extent interactions between different medications, life-style and inter-patient variability affect this association^4^. It is conceivable that the metabolic effects of antipsychotics are partly mediated by genetic predisposition. So far, studies on the genetics of antipsychotic response have been small (*n* < 400) and unable to identify replicating associations^9,10^.

In addition, there is evidence that the increased prevalence of T2D in patients with SCZ is not purely medication induced: Proteomic studies have revealed perturbed expression of genes involved in glucose metabolism in brain tissue and elevated insulin levels in peripheral blood of first-episode SCZ patients compared to controls^11,12^. More recently, a large study following over 2.5 million Danish individuals found that antipsychotic-naïve SCZ patients were three times more likely to develop T2D than the general population, with antipsychotic drug use further increasing that risk^13^. This, along with findings from a systematic review and meta-analysis^14^, suggests that impaired glucose homeostasis may already be present in drug-naïve SCZ patients.

It is also plausible that the observed overlap between SCZ and T2D is due to common susceptibility variants^2^. Both diseases are highly polygenic, and genome-wide association studies (GWAS) to date have successfully identified a substantial number of risk loci for T2D^15–18^ and SCZ^19–21^. Functional analyses showed that risk variants for SCZ are enriched for enhancers mapping to pancreatic beta cells^19^, and that variants associated with BMI – a key risk factor for T2D – predominately map to central nervous system pathways^22^. Genetic research into the shared pathobiology of SCZ and T2D has been limited to date, and has mainly focused on patients with one of the two disorders^2^. If SCZ without T2D comorbidity and SCZ with T2D are partly underpinned by different genetic aetiologies, such study designs will fail to identify risk factors predisposing to the latter.

Here, we investigate the presence of shared genetic risk factors for T2D and SCZ using genotype data from a novel cohort comprising three patient groups (T2D only, SCZ only, and comorbid SCZ and T2D), as well as summary data from large-scale disease-specific GWAS. First, we conduct genome-wide comparisons between all three patient groups, as well as population controls; next, we assess the genetic overlap between the two disorders using polygenic risk scores; finally, we use summary statistics from published GWAS to search for genetic risk factors shared between SCZ and T2D.

## Methods

### Data sets

The GOMAP (Genetic Overlap between Metabolic and Psychiatric disorders) study comprises a collection of 2,747 DNA samples from four different patient categories: T2D patients, SCZ patients, individuals with both SCZ and T2D (referred to from here on as SCZplusT2D), and individuals with a different psychiatric diagnosis (this last group is not used in further analyses reported here) (Table 1). SCZ patients with and without T2D were recruited at the Dromokaitio Psychiatric Hospital and Dafni Psychiatric Hospital in Athens. SCZ diagnosis was determined by structured clinical interview of the Diagnostic and Statistical Manual of Mental Disorders 4^th^ edition (DSM-IV)^23^. T2D participants were recruited from diabetes outpatient clinics at Hippokrateio General Hospital and Laiko General Hospital. T2D status was assessed in all participants based on criteria outlined by the American Diabetes Association^24^. All participants gave written informed consent.

**Table 1:** Sample numbers in the three phenotype groups in GOMAP before and after QC

Sample group	Pre-QC	Post-QC
SCZ	977	924
T2D	885	822
SCZplusT2D	542	505
Other	343	331
Total	2747	2582

### Quality control

A total of 2,474 samples and 538,448 markers were successfully genotyped on the Illumina HumanCoreExome 12v1.0 BeadChip (Illumina, San Diego, CA, USA) at the Wellcome Trust Sanger Institute, Hinxton, UK. Quality control (QC) of genotype data was performed following a standard protocol^25^ using the PLINK^26^ software package. Individuals were removed if they had a call rate below 90%, discordant values for genotyped and reported sex or had heterozygosity rates deviating more than three standard deviations from the mean. For duplicates and related sample pairs (pi_hat > 0.2), we excluded one and retained the other.

In order to identify potential ethnic outliers, we performed multidimensional scaling (MDS) on a merged dataset comprising GOMAP and three other Greek sample collections: TEENAGE^27^, a collection of adolescents from the general Greek population, HELIC-POMAK^25^ and HELIC-MANOLIS^28^, two Greek isolated population cohorts. We removed seven individuals from GOMAP as outliers based on the first and second MDS components (Supplementary Figure 1).

A total of 2,582 samples passed QC (Supplementary Table 1; samples size of each diagnostic category: SCZ, *n* = 924; T2D, *n* = 822; T2D/SCZ, *n* = 505; other diagnosis, *n* = 331).

After removal of individuals failing QC, variants were filtered for call rates lower than 98%, a Hardy-Weinberg Equilibrium deviation *p* value < 1 × 10^–4^ and cluster separation scores below 0.4. In addition, we removed X-chromosomal markers not within the pseudo-autosomal region with heterozygous haploid genotypes in males. A total of 524,271 autosomal and X-chromosomal markers passed QC (Supplementary Table 2).

Since GOMAP is a cases-only sample collection, we selected two independent Greek cohorts, TEENAGE^29^ (*n* = 413) and ARGO (*n* = 712), as control data sets. ARGO comprises osteoarthritis cases and healthy controls from Larisa, Greece. Samples from all three cohorts formed a cluster in MDS analysis (Supplementary Figure 2).

### Imputation

Following QC we merged GOMAP with 413 samples from TEENAGE^27^ and 712 from ARGO, an in-house Greek sample collection. We performed pre-phasing of the merged dataset in SHAPEIT^30^ and imputed the phased haplotypes with IMPUTE2^31^ using a combined reference panel consisting of UK10K^32^, 1000 Genomes^33^ and HELIC-MANOLIS^28^. We filtered imputed genotypes for Hardy-Weinberg equilibrium deviation (*p* value < 1 × 10^-4^), IMPUTE2 info scores < 0.4, and a minor allele frequency (MAF) > 1%. A total of 14,528,340 markers passed imputation QC.

### GWAS

We carried out a GWAS for each case-case and case-control combination in GOMAP using the ‘method --expected’ option, which performs an additive association test, adjusting for the first ten MDS components using SNPTEST version 2.5^34^.

### Genetic risk scores

We constructed polygenic risk scores for T2D and SCZ in GOMAP based on effect size estimates from the DIAGRAM and PGC consortium, respectively. The risk score analyses are divided into two stages: first, we constructed using only established risk variants for each diseases; next, we relaxed our inclusion criteria incrementally by using all variants falling below a given *p* value threshold.

#### Established risk variants

For SCZ, we obtained odds ratios (ORs) of 125 autosomal risk variants from the psychiatric genomics consortium (PGC)^19^ (Supplementary Table 3). We excluded three X-chromosomal markers of the original 128 independent variants identified by Ripke et al^19^. as calculating scores for non-autosomal alleles is not straightforward.

We used 73 variants identified in a trans-ethnic meta-analysis^15^ for the T2D risk score. In order to match the ancestry of the base data as closely to GOMAP as possible, we looked up summary statistics of all independent variants (76 in total) identified in the trans-ethnic study^15^ in the DIAGRAMv3 stage 1 meta-analysis^17^(Supplementary Tables 4) based on samples of European descent. Three of the 76 variants were not present in the DIAGRAMv3 data and therefore excluded.

We used PRSice version 1.25^35^ to calculate the risk scores in GOMAP and test for an association between scores and phenotype. Briefly, for each variant the number of risk alleles in the target data (GOMAP) is multiplied by the log(OR) from the base data (DIAGRAM or PGC). The total score for an individual is the average score across all SNPs in the set. Following the approach described by Purcell et al.^36^, two logistic regression models are used to obtain the variance in phenotype explained (Nagelkerke’s pseudo *R*^2^):

Full model:

Phenotype ~ Score + C1 + C2 + C3 + C4 + C5 + C6 + C7 + C8 + C9 + C10

Null model:

Phenotype ~ C1 + C2 + C3 + C4 + C5 + C6 + C7 + C8 + C9 + C10

In the full model, phenotypes are regressed on risk scores adjusting for the first ten multi-dimensional scaling (MDS) components; in the null model, phenotypes are regressed on MDS components only. Most, but not all studies contributing to the DIAGRAMv3 meta-analysis adjusted for sex; conversely, sex was not adjusted for in the individual GWAS included in the PGC-SCZ meta-analysis. We therefore decided not to add sex as a covariate in our risk score model. The final pseudo *R*^2^ estimate is obtained by:

$$R^2_{{\rm{final}}} = R^2_{{\rm{full}}}-R^2_{{\rm{null}}}$$

A *p* value for association of score with phenotype was obtained from the full model. Risk score analysis was carried out in each pairwise comparison between the three disease groups and controls in GOMAP.

To assess whether the sample size difference between the single-disease and comorbid group in GOMAP affects the strength of the risk scores, we randomly down-sampled the SCZ-only and T2D-only group to 500 individuals each and performed risk score analyses with this reduced set. We repeated this process 5,000 times and computed average pseudo *R*^2^ and *p* values.

#### Genome-wide risk scores

In addition to calculating risk scores based on established genome-wide significant risk variants, we performed polygenic scoring as implemented in PRSice^35^, a pipeline automating data preparation in PLINK^26^ and risk score regression in R. First, *P* value informed linkage disequilibrium (LD) clumping was performed on the intersection of variants between the base summary statistics (DIAGRAM^17^ and PGC^19^) and target data (GOMAP), using an r^2^ threshold of 0.1 and a window size of 250 kb. Next, alleles are matched between the base and target data and ambiguous variants are removed to produce a final list of clumped variants used for the risk scores. Score calculations and regression analyses are conducted following the same procedure as outlined for the established risk variants. We performed risk score analyses at ten cumulative *p* value thresholds: *p* < 5 × 10^−8^, *p* < 0.001, *p* < 0.005, *p* < 0.05, *p* < 0.1, *p* < 0.2, *p* < 0.3, *p* < 0.4, *p* < 0.5, *p* < 1; all variants below a given threshold in the base data were included in the score.

### Summary statistics-based overlap analyses

We obtained genome-wide summary data for T2D from the DIAGRAMv3 meta-analysis^17^, and for SCZ from the Psychiatric Genomics Consortium (PGC) meta-analysis^19^. To assess the genetic overlap between the two data sets, we performed three complementary analyses, which have been described previously^37^ and are briefly outlined below.

#### LD score regression

LD score regression relies on the assumption that variants in strong LD with a causal variant will have a higher association statistic than variants in low LD. When comparing the effect estimates of two GWAS, LD score regression can be used to compute the overall (i.e. genome-wide) correlation between them.

We performed LD-score regression^38^ on the DIAGRAMv3^17^ and PGC^19^ summary statistics, using LD scores computed on the 1000 Genomes European sample^39^.

#### Regional colocalisation test

We performed a Bayesian colocalisation analysis^40^ to test for the presence of association signals in distinct blocks across the genome. At each region the model uses Z-scores and standard errors from two association studies to generate posterior probabilities for each of five hypotheses:

##### Hypothesis 0:

No causal variants

##### Hypothesis 1:

One causal variant for disease 1.

##### Hypothesis 2:

One causal variant for disease 2.

##### Hypothesis 3:

One causal variant for disease 1 and 2.

##### Hypothesis 4:

One causal variant each for disease 1 and 2.

Splitting the genome into uniform segments without accounting for LD structure can result in the double-counting of signals if segment boundaries happen to fall within an associated region. We downloaded LD-blocks pre-computed using the LDetect algorithm^41^ and the European sample of the 1000 Genomes Phase 1 data^33^ (https://bitbucket.org/nygcresearch/ldetect-data). The output of the test includes posterior probability estimates, as well as the highest absolute Z-score for each phenotype in a given region. We followed up regions with a high (≥0.9) posterior probability for either hypothesis 3 or 4 by taking the variants corresponding to the highest absolute Z-scores in DIAGRAMv3 and PGC-SCZ and querying their function and closest protein coding genes using the Ensembl REST API^42^.

#### Extent of shared signals

To assess the extent of association signals common between DIAGRAMv3 and PGC-SCZ, we took all variants present in both data sets and performed *p* value informed LD pruning (*r*^2^ > 0.1) to obtain an independent set. We constructed 2 × 2 contingency tables of overlapping variants at ten cumulative *p* value thresholds (*P*_t_) by counting how many variants fell above and below each P_t_ in DIAGRAMv3 and PGC-SCZ. We then tested for an excess of shared signals at each *P*_t_ by applying a *χ*^*2*^ test, which gives an overlap *p* value. Empirical overlap *p* values were obtained by randomly permuting the GWAS *p* values in each data set 1,000,000 times and repeating the test on each permuted set.

#### Gene and pathway analysis

We used MAGMA^43^ to perform gene and pathway analyses on the DIAGRAMv3 and PGC summary statistics separately. We annotated variants in each dataset to genes according to dbSNP version 135 coordinates and NCBI 37.3 gene definitions. We allowed for a 20 kilobase (kb) window around the transcription start and stop sites to also include proximal regulatory elements. We combined the results of the gene-level analysis into biological pathways using gene-set definitions from two comprehensive databases: the Molecular Signatures Database canonical pathways collection^44^, comprising 1,329 manually curated gene-sets, and the Gene Ontology pathway database^45^, comprising 6,166 automatically annotated gene-sets. Significance was defined as a false discovery rate (FDR) corrected *p* value (*q* value) < 0.05.

## Results

### GWAS

We performed six case-case and case-control genome-wide association studies in GOMAP and population controls (Supplementary Figures 6-9). There was no indication of inflation of test statistics, with lambda values ranging from 0.99 to 1.04 (Supplementary Figures 8-9).

We identified two genome-wide significant signals in the SCZplusT2D vs controls analysis (Table 2; Supplementary Figure 6c). The most strongly associated variant resides within an intron of the *PACRG* gene (chr6:163319442_G/A, effect allele (EA) G, effect allele frequency (EAF) 0.91, OR 3.81 [95% CI: 3.32–4.29], *p* value = 5.46 × 10^−9^). The second signal is located in an intron of *RP11-587H10.2* on chromosome 8 (rs1449245, EA A, EAF 0.79, OR 1.96 [95% CI 1.77–2.20], *p* value = 2.58 × 10^−8^).

**Table 2:** Top variant of genome-wide significant signals in the GOMAP GWAS analyses

Variant	GWAS	EA	NEA	EAF	OR (95% CI)	Info	*P* value
chr6:163319442	SCZplusT2D vs Controls	G	A	0.91	3.81 (3.32–4.29)	0.56	5.46E-09
rs1449245	SCZplusT2D vs Controls	A	G	0.79	1.96 (1.71–2.2)	0.85	2.58E-08
rs7903146	T2D vs Controls	T	C	0.38	1.66 (1.5–1.81)	1.00	3.31E-11
rs7903146	T2D vs SCZ	C	T	0.61	1.53 (1.39–1.67)	1.00	1.09E-09
rs17616243	SCZ vs Controls	T	C	0.16	2.03 (1.79–2.27)	0.72	3.26E-09
rs6598475	T2D vs Controls	T	G	0.36	1.56 (1.4–1.72)	0.93	1.95E-08

*EA* effect allele, *NEA* non-effect allele, *EAF* effect allele frequency, *OR* odd ratio, *CI* confidence interval

Three further signals reached genome-wide significance in other analyses (Table 2): an intronic single nucleotide polymorphism (SNP) in *TCF7L2* (rs7903146, EA T, EAF 0.38), a well-established T2D risk gene^17^, in the T2D vs controls (OR 1.66 [95% CI 1.50-1.80], *p* value = 3.31 × 10^−11^) and T2D vs. SCZ analyses (OR 1.53 [95% CI: 1.39–1.67], *p* value = 1.09 × 10^−9^); an intronic SNP in *BMPR1B* (rs17616243, EA T EAF 0.16, OR: 2.03 [95% CI: 1.79–2.27], *p* value = 3.26 × 10^−9^) in the SCZ vs. controls GWAS; and an intronic SNP in *PCSK6* in the T2D vs controls GWAS (rs6598475, EA T, EAF 0.36, OR: 1.56 [95% CI: 1.40–1.72], *p* value = 1.95 × 10^−8^). (Table 3)

**Table 3:** Overlap analysis between DIAGRAM and PGC summary statistics

*P*_*t*_ value	Variants	*χ* ^2^	*P* value	*P*_perm_ value
0.5	58504	1.4	2.30E-01	2.32E-01
0.1	6247	39.7	3.00E-10	0.00E + 00
0.05	2324	40.9	1.60E-10	0.00E + 00
0.04	1749	53.5	2.50E-13	0.00E + 00
0.03	1180	49	2.50E-12	0.00E + 00
0.02	658	32.4	1.30E-08	0.00E + 00
0.01	287	41.4	1.30E-10	0.00E + 00
0.005	125	37.7	8.10E-10	0.00E + 00
0.001	19	14.2	1.70E-04	8.30E-04
5.00E-04	10	13.8	2.00E-04	2.00E-03

For each *p* value threshold (*P*_t_) the number of independent variants overlapping at this threshold is given, along with the resulting chi-squared statistic (*χ*^2^), *p* value (*P*) and empirical *p* value obtained by permutations (*P*_perm_).

### Genetic risk scores

**Fig. 1:**
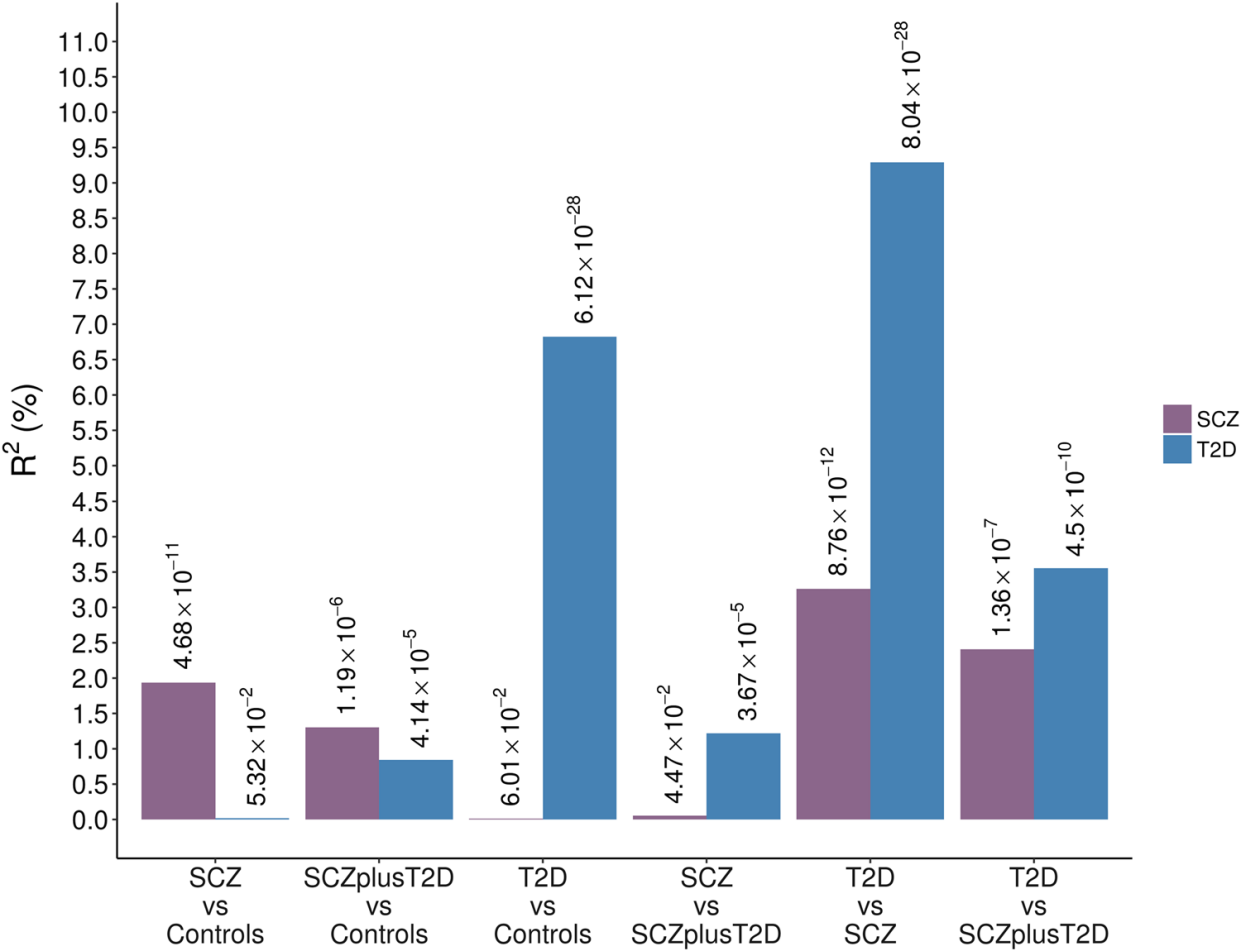
Genetic risk scores of established risk variants for SCZ and T2D in GOMAP. For each analysis Nagelkerke’s pseudo *R*^2^ values are plotted and *p* values for association between score and phenotype are denoted above each bar

We performed genetic risk score analyses of SCZ and T2D for each pairwise case-case and case-control combination in GOMAP (Fig. 1). In the case-control analyses, risk scores for SCZ and T2D were significantly associated with these respective disorders (SCZ *R*^2^ = 1.7%, *p* value = 5.25 × 10^−9^; T2D *R*^2^ = 6.8%, *p* value = 6.12 × 10^−27^), serving as a positive control for the validity of the included variants and patient groups. Conversely, risk scores for one disorder were not associated with the other in the case-control comparisons. In the comorbid sample both SCZ and T2D risk scores were significantly associated with phenotype (SCZ risk score *p* value = 7.17 × 10^−5^; T2D risk score *p* value = 4.14 × 10^−4^), with *R*^2^ values lower than those in the single-disease groups (SCZ risk score *R*^2^ = 1%; T2D *R*^2^ = 0.8%).

**Fig. 2:**
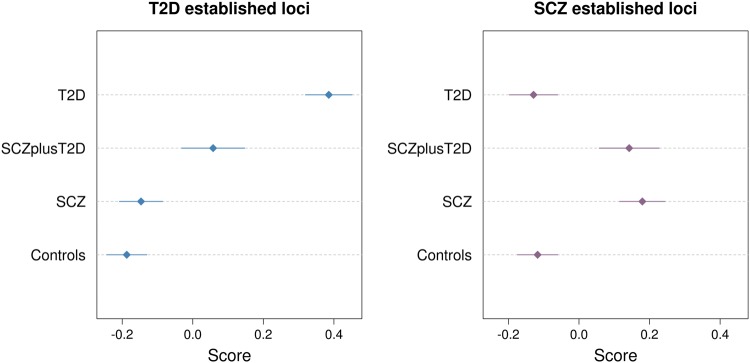
Mean and 95% confidence intervals of standardised risk scores for established SCZ and T2D loci in each sample group in GOMAP. Risk scores were constructed based on the effect sizes of 73 and 125 variants from DIAGRAMv3 and PGC-SCZ, respectively.

In the comparison between T2D and SCZ cases, risk scores for T2D explained 9.3% of variance (*p* value = 8.04 × 10^−28^) and risk scores for SCZ explained 3.4% of variance (*p* value = 8.06 × 10^−12^). These R^2^ values may be higher than in the case-control analyses due to the fact that controls are population based and not ascertained for either SCZ or T2D status; it is therefore plausible that a subset of controls carries risk alleles for these disorders. In the comparison of individuals with SCZ to those with SCZ and T2D, SCZ risk scores and their *R*^2^ values were not significantly associated with disease. This is expected, as both sample groups are likely to be enriched for SCZ risk alleles. Interestingly, the R^2^ estimate of the T2D variant risk scores in the T2D vs. SCZplusT2D analysis was intermediate in magnitude to that measured in the SCZ vs. SCZplusT2D and the SCZ vs T2D analyses. This can be recapitulated by examining the average T2D scores across the different sample groups (Fig. 2): the average score of the SCZplusT2D sample is higher than for the SCZ-only sample but lower than for the T2D-only sample, indicating that the comorbid group is enriched for T2D risk alleles compared to the SCZ-only group.

To determine whether the observed strength of association of the risk scores was influenced by the difference in sample size among the single-disease and comorbid groups, we repeated the risk score analyses with equally-sized (*n* = 500), randomly down-sampled T2D- and SCZ-only cases. Risk scores significantly associated with phenotype using the full data set remained significant even with the decreased sample size (*p* < 0.05) (Supplementary Figure 3).

It has been shown that the inclusion of variants not reaching genome-wide significance can enhance the power of genetic risk scores^36^. We constructed polygenic scores at ten cumulative *p* value thresholds using the same base data sets (DIAGRAMv3 and PGC-SCZ) as for the established variant scores. For the SCZ scores, the most stringent threshold (*p* < 5 × 10^-8^) resulted in lower levels of association and pseudo-R^2^ estimates than the established variant score, due to the fact that some of the variants included in the latter had *p* > 5 × 10^-8^ in the PGC-SCZ discovery data, which was used here, and were therefore excluded. At more permissive *p* value thresholds the strength of association increased by several orders of magnitude compared to the established variant scores for all but the SCZ vs SCZplusT2D and T2D vs Controls analyses (Supplementary Figure 4). While pseudo-R^2^ also increased at the first increments variant inclusion, they plateaued or even decreased slightly for thresholds with *p* > 0.005. While more relaxed thresholds will include more variants with true effects, they will inevitably also add more null variants contributing to noise.

Unlike the SCZ score, T2D scores demonstrated decreasing levels of association as more variants were included in the risk score (Supplementary Figure 5). This can be explained by the fact that only 21 of the 73 variants used for the established loci score were retained after LD clumping, again due to their strength of association in the DIAGRAMv3 discovery data. In total, only 15 variants were included at the most stringent *p* value threshold, whereas over 1,000 were used at *p* < 0.001. As a result, the ‘signal-to-noise’ ratio will have increased drastically, explaining the sharp drop in pseudo-*R*^2^ values.

### Summary statistics-based overlap analyses

We investigated the genetic overlap between summary data from the DIAGRAMv3 meta-analysis for T2D^17^ and the PGC meta-analysis for SCZ^19^ using both genome-wide and regional approaches.

#### LD score regression

There was no significant correlation between these data sets on a genome-wide scale (*r*^2^ = −0.01, SE = 0.04, *p* value = 0.82; Supplementary Methods), as previously reported elsewhere^38^.

#### Colocalisation analysis

We employed a Bayesian colocalisation analysis to search for genomic regions that potentially exert pleiotropic effects. For each region, the method returns posterior probabilities for the five tested hypotheses, as well as the maximum absolute Z-scores found in each of the two input data sets; in some cases, there is more than one variant with the same Z-score (i.e. effect estimate) in a region.

There were no regions with a high posterior probability (>0.9) of containing one causal variant common to both diseases. However, five regions had a high posterior probability of harbouring two distinct causal variants (Supplementary Table 3).

The first of these regions is located on chromosome 2 and includes nominally significant SCZ variant (top variant in PGC: rs10189857, *p* = 5.14 × 10^−7^)^19^ in an intron of *BCL11A*, and a T2D risk locus upstream of the same gene (top variant in DIAGRAMv3: rs243021, *p* = 3 × 10^−15^)^46^.

The second region falls within the major histocompatibility complex on chromosome 6, which is known to harbour several SCZ and T2D loci^17,19^. There were three variants with the same effect size for T2D, one of which lies in an intron of *SLC44A* (rs9267658, OR 0.89, 95% CI 0.85-0.94, *p* = 2.2 × 10^-5^). The strongest SCZ signal occurred at rs3117574 (OR 0.85, 95% CI 0.82-0.89, *p* = 6.71 × 10^−19^), a variant in the 5’ untranslated region of *MSH5*, a protein involved in meiotic recombination and DNA mismatch repair.

The third region resides on chromosome 7, harbouring both a known T2D locus downstream of *KLF14* (top variant in DIAGRAMv3: rs10954284, *p* = 1.20 × 10^−8^) and a known SCZ variant at rs7801375 (PGC *p* = 2.26 × 10^−8^)^19^.

The fourth region, identified on chromosome 8, does not contain any known T2D or SCZ associated variants. The strongest signals in that region occur at rs11993663 for SCZ (PGC *p* = 1.46 × 10^−7^) and rs17150816 for T2D (DIAGRAMv3 *p* = 1.60 × 10^−5^).

Finally, a region identified on chromosome 15 encompasses a known SCZ locus in the *VPS13C* gene (top variant in PGC: rs12903146, *p* = 3.00 × 10^−10^), as well as the *C2CD4A-C2CD4B* locus, which has been associated with T2D in East Asian populations and also replicated in Europeans (top variant in DIAGRAMv3: rs8026735, *p* = 2.50 × 10^−7^)^47^.

#### Extent of shared signals

We assessed the extent of shared association signals between DIAGRAMv3 and PGC-SCZ at ten different p-value thresholds (*P*_t_) and found significant evidence for overlap (*p*_perm_ < 0.05) at all but one P_t_ (Table 2). Of the 19 variants overlapping at *P*_t_ = 0.001, five are located in known T2D loci, and four within known SCZ loci. One of the variants identified at this *P*_t_, rs6488868, is a synonymous SNP in *SBNO1*, and in partial LD with both a known T2D (rs1727313, *r*^2^ = 0.53) and a known SCZ (rs2851447, *r*^2^ = 0.45) risk variant. The two risk variants lie in the 3’UTR and in an intron of *MPHOSPH9*, respectively, and are also in LD with each other (*r*^2^ = 0.79). Other variants fall within or around several genes previously linked to SCZ or T2D, such as *CACNA1*, *HLA-B*, *PROX1* and *BCL11A*^17,19^ (Supplementary Table 4).

#### Gene and pathway analysis

We tested for enrichment of association signals in genes and pathways in the DIAGRAM and PGC summary statistics. We did not identify any pathways that were significantly associated (*q* value < 0.05) with both SCZ and T2D. In the gene-level analysis, 29 genes had a *q* value < 0.05 in both data sets (Supplementary Table 5). Ten of the genes have been previously associated with SCZ and/or T2D. Of note, variants in or in close proximity to *ZFAND6*, *PROX1*, and *HLA-B* were also found to overlap at *P*_t_ = 0.001. *SLC44A4*, which is strongly associated with SCZ (*q* value = 4.73 × 10^−11^), falls within the region on chromosome 6 identified in the colocalisation analysis.

## Discussion

We investigated the genetic overlap between SCZ and T2D, using summary statistics from large-scale meta-analyses and genome-wide genotype data from a dedicated collection of individuals with SCZ, T2D or both disorders. The work presented here benefits from clinically ascertained diagnoses and robust base data sets used to construct the risk scores. Due to the limited sample size and, consequently, low power to detect genetic associations in GOMAP, we did not expect to identify novel genome-wide significant loci, but rather to harness the comorbid patient group for risk score analyses. The two genome-wide significant signals identified in the SCZplusT2D vs controls GWAS map to introns of *PACRG* and *RP11-587H10.2*. *PACRG* has been associated with the risk of leprosy^48^, while *RP11-587H10.2*, a long non-coding RNA, is of unknown function. Replication of these signals in independent data sets is required to establish or refute them as novel associations.

Our main finding arises from the risk score analyses, which demonstrated that the SCZplusT2D sample is enriched for both SCZ and T2D risk alleles compared to controls, in line with the increased prevalence of T2D among schizophrenia patients being at least partly due to genetic predisposition^2,3^. Patients suffering from both diseases had SCZ risk scores comparable to the SCZ-only group but fell between the SCZ-only and T2D-only groups for T2D risk scores. This implies that patients with comorbid SCZ and T2D carry almost the same SCZ risk allele profile as SCZ patients without T2D but have fewer of risk-increasing variants for T2D than T2D patients without comorbid SCZ. Two conclusions might be drawn from this: first, at least part of the risk for T2D in SCZ patients is driven by genetic predisposition to T2D, rather than antipsychotic use alone; and second, the comorbid group appear to have a less strong T2D genetic risk profile compared to T2D-only patients. This is in line with environmental factors, including response to antipsychotic treatment and sedentary lifestyle, contributing to T2D risk. Such factors might exacerbate an otherwise moderate genetic predisposition to T2D.

To our knowledge, three other studies have to date compared risk scores for T2D and SCZ^36,49,50^. Purcell et al. first performed SCZ risk scores analysis in a T2D sample but did not identify a significant correlation between scores and phenotype^36^, potentially due to the relatively low sample sizes available at the time (~3300 cases for SCZ; ~1900 cases for T2D). More recently, a study investigating the genetic liability to SCZ in immune-related disorders found a weak association between SCZ risk scores and T2D^50^. The investigators used an earlier release of the PGC-SCZ summary data^20^ with lower sample numbers than currently available. One study has previously reported an association between T2D risk scores based on DIAGRAM summary statistics and self-reported diabetes (any type) in individuals with psychosis, but did not detect an association when repeating the analysis for SCZ risk scores^52^.

The SNP-based overlap analysis highlighted one region where a known T2D and a known SCZ signal map to the same locus in the *MPHOSPH*9 gene^15,19^, which encodes a phosphoprotein highly expressed in the cerebellum. This gene has been previously associated with multiple sclerosis;^51^ however, its function is not well understood. We also identify *PROX1* as a potentially pleiotropic locus based on the gene analysis and the SNP-based overlap test. *PROX1* has been previously implicated in each of T2D and SCZ, and acts both as a transcriptional activator and repressor depending on the cellular context. It has been implicated in murine beta-cell development^52^, as well as in neurogenesis in humans^53^. One possible explanation for the cross-phenotype associations of these loci might be that they influence T2D and SCZ by acting in different biological pathways. However, follow-up in functional (e.g. expression or proteomic) data is needed in order to evaluate this hypothesis.

In this study, we have shown that genetic predisposition to SCZ and genetic predisposition to T2D are both associated with comorbidity. Future studies with larger sample sizes and detailed phenotype information (ideally including longitudinal medication data) will be necessary to precisely disentangle the shared genetic basis of SCZ and T2D.

## Electronic supplementary material

Supplementary Material

## Data Availability

Genetic data for the GOMAP study has been deposited at the European Genome-Phenome Archive (EGA) which is hosted at the EBI and the CRG, under accession number EGAS00001002723.
