# Supplementary material for: Evidence for genetic contribution to the increased risk of type 2 diabetes in schizophrenia
Source: Transl Psychiatry. 2018 Nov 23;8:252. doi: 10.1038/s41398-018-0304-6 (PMC6251918; doi:10.1038/s41398-018-0304-6)
Supplement: Supplementary file 1 — Supplementary Material [file 41398_2018_304_MOESM1_ESM.docx]

**Evidence for shared genetic aetiology between type 2 diabetes and schizophrenia**

**Supplementary Data**

Supplementary Figures 2

Supplementary Tables 11

**Supplementary Figure 1. Multidimensional scaling analysis pre-QC**

**
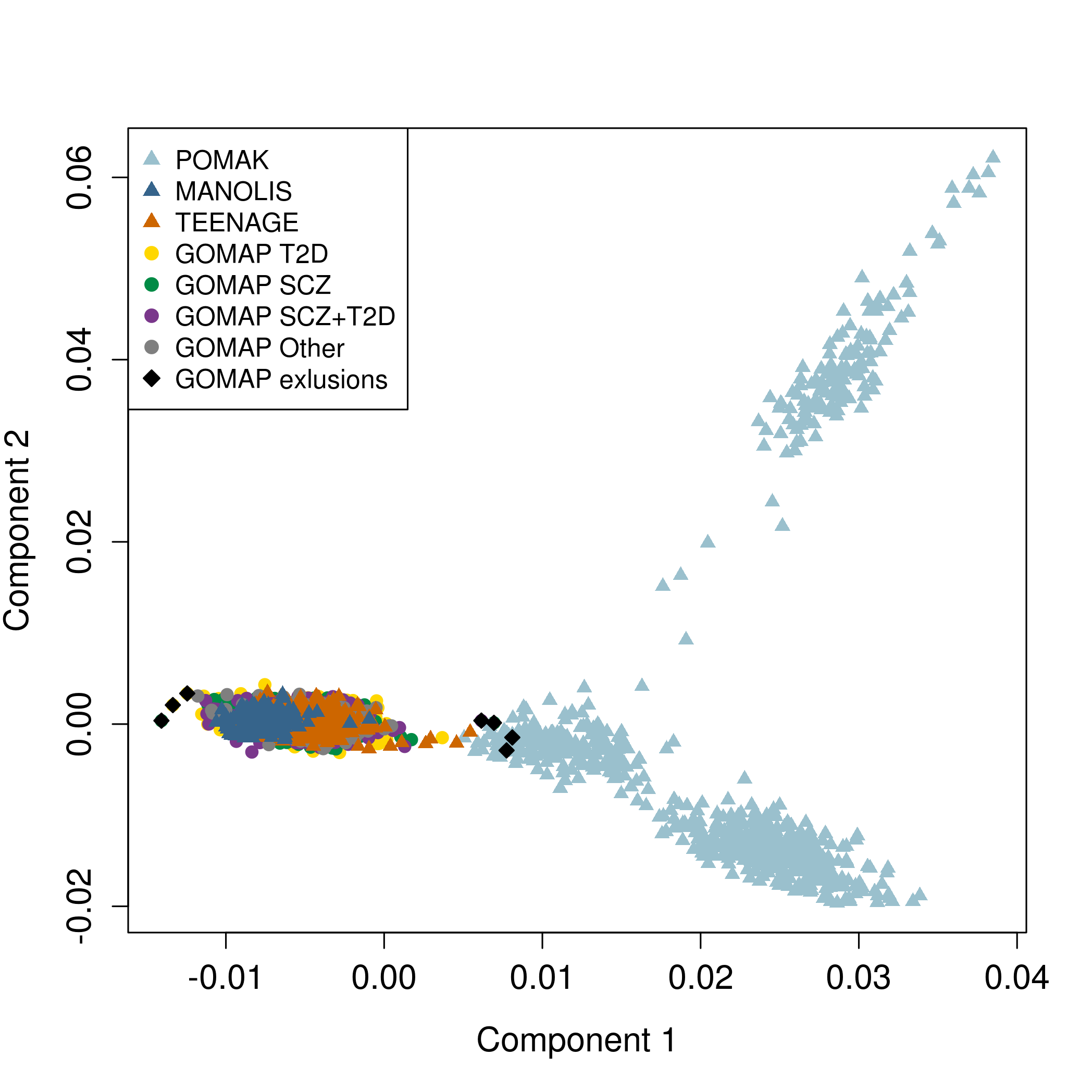
**

Components 1 and 2 from multidimensional scaling (MDS) analysis of GOMAP (pre-QC), HELIC-POMAK, HELIC-MANOLIS and TEENAGE. Each data point represents one individual. Black diamond shapes depict individuals excluded as ethnic outliers.

**Supplementary Figure 2. Multidimensional scaling analysis post-QC**

**
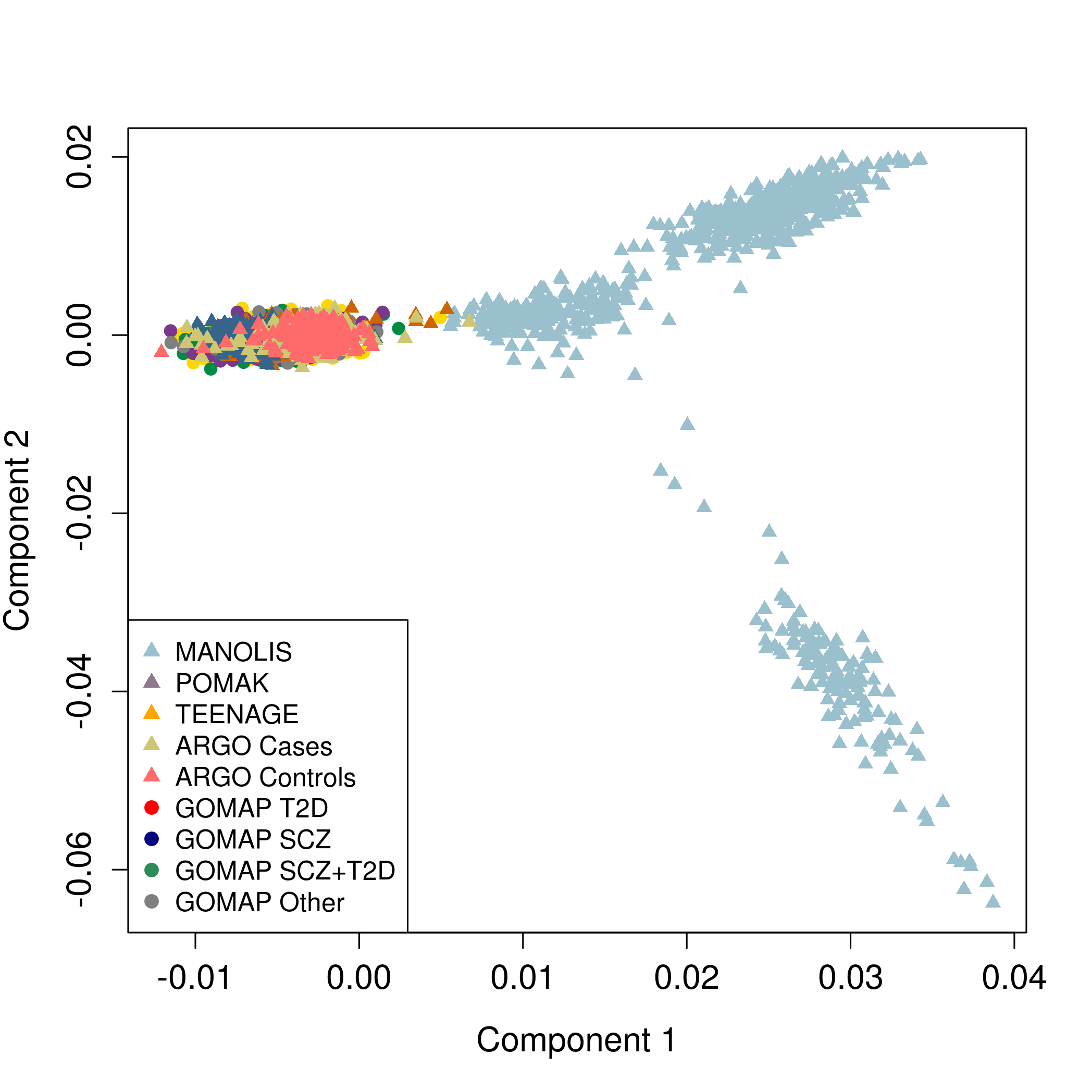
**

Components 1 and 2 from MDS analysis of GOMAP (post-QC), HELIC-POMAK, HELIC-MANOLIS, ARGO and TEENAGE. Each data point represents one individual.

**Supplementary Figure 3. Genetic risk scores for established T2D and SCZ risk loci in GOMAP**

**
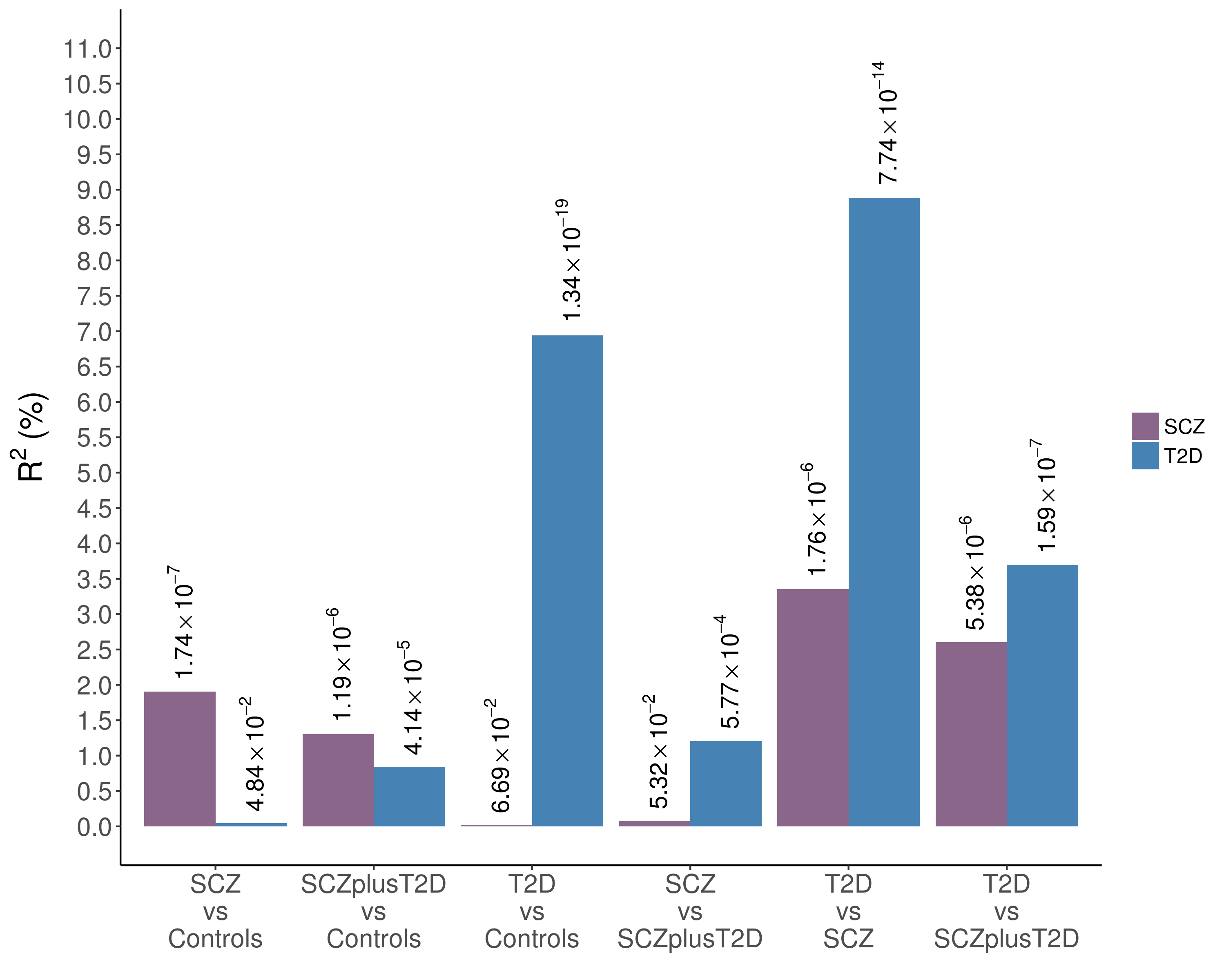
**

The SCZ and T2D groups were each downsampled to 500 cases and the risk scores calculated in this reduced set. The results shown are average p-values and pseudo-R^2^ values of repeating the downsampling step 5,000 times.

**Supplementary Figure 4. Polygenic risk score analyses for SCZ in GOMAP**

**
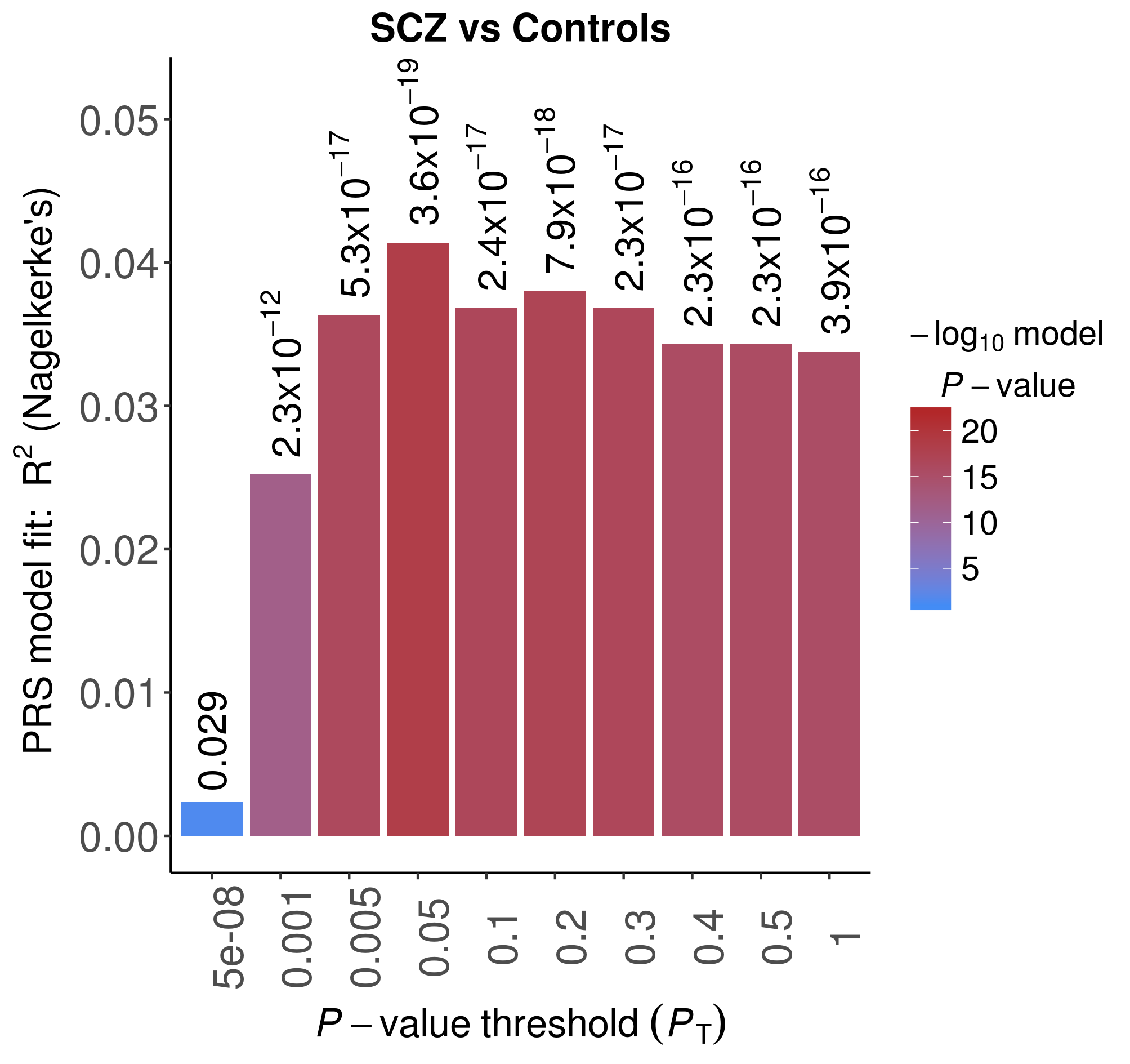

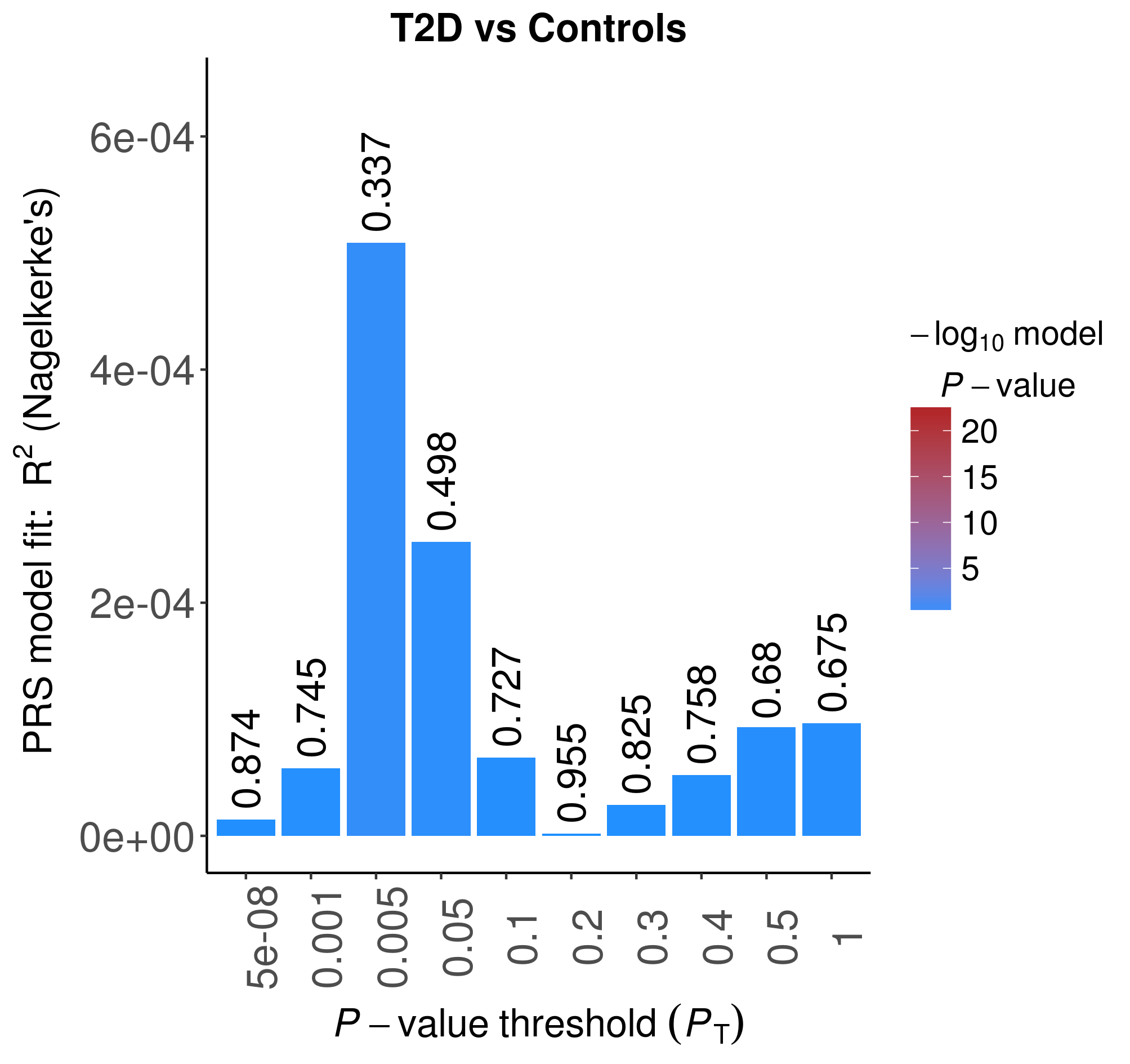
**

**
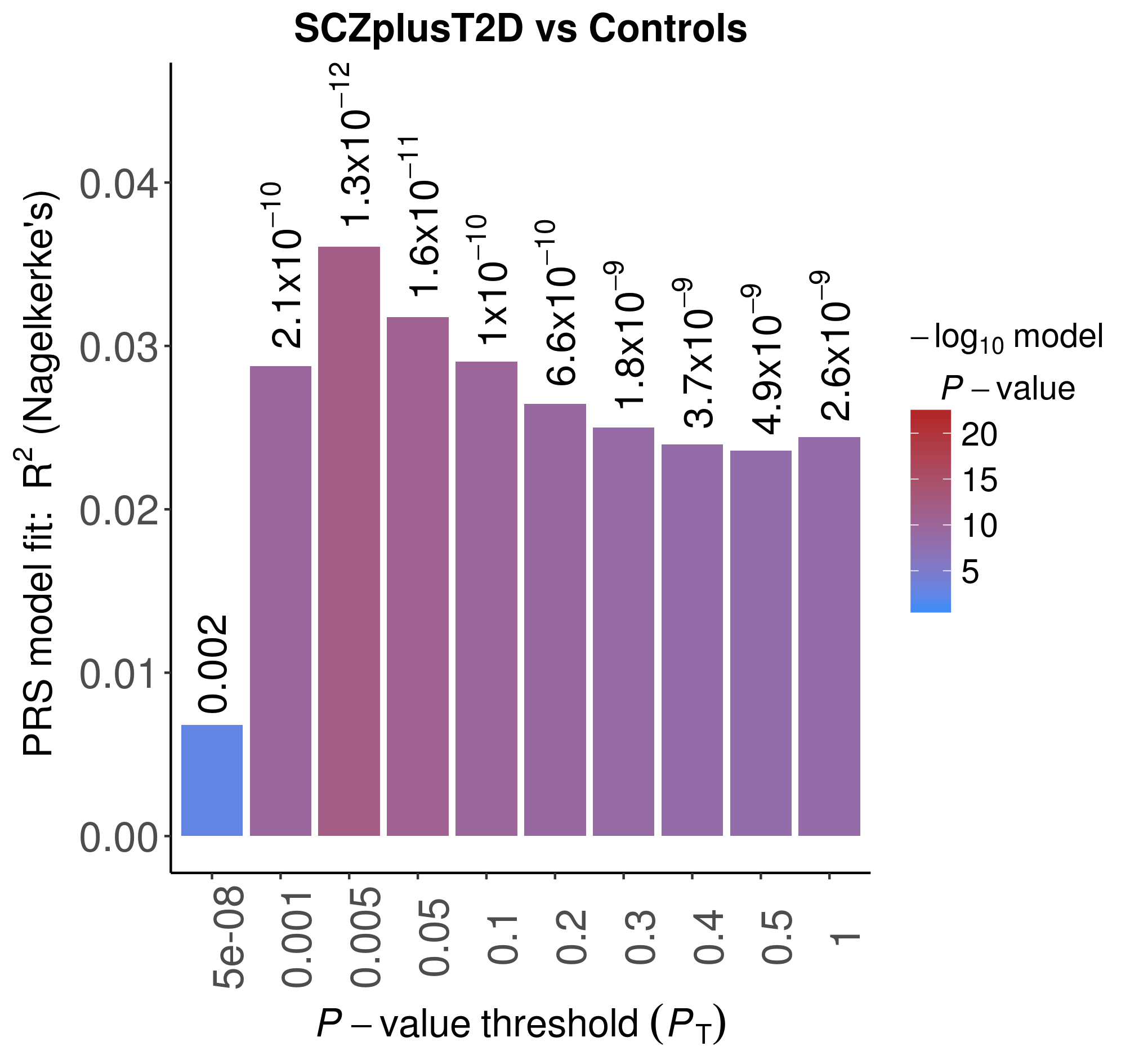

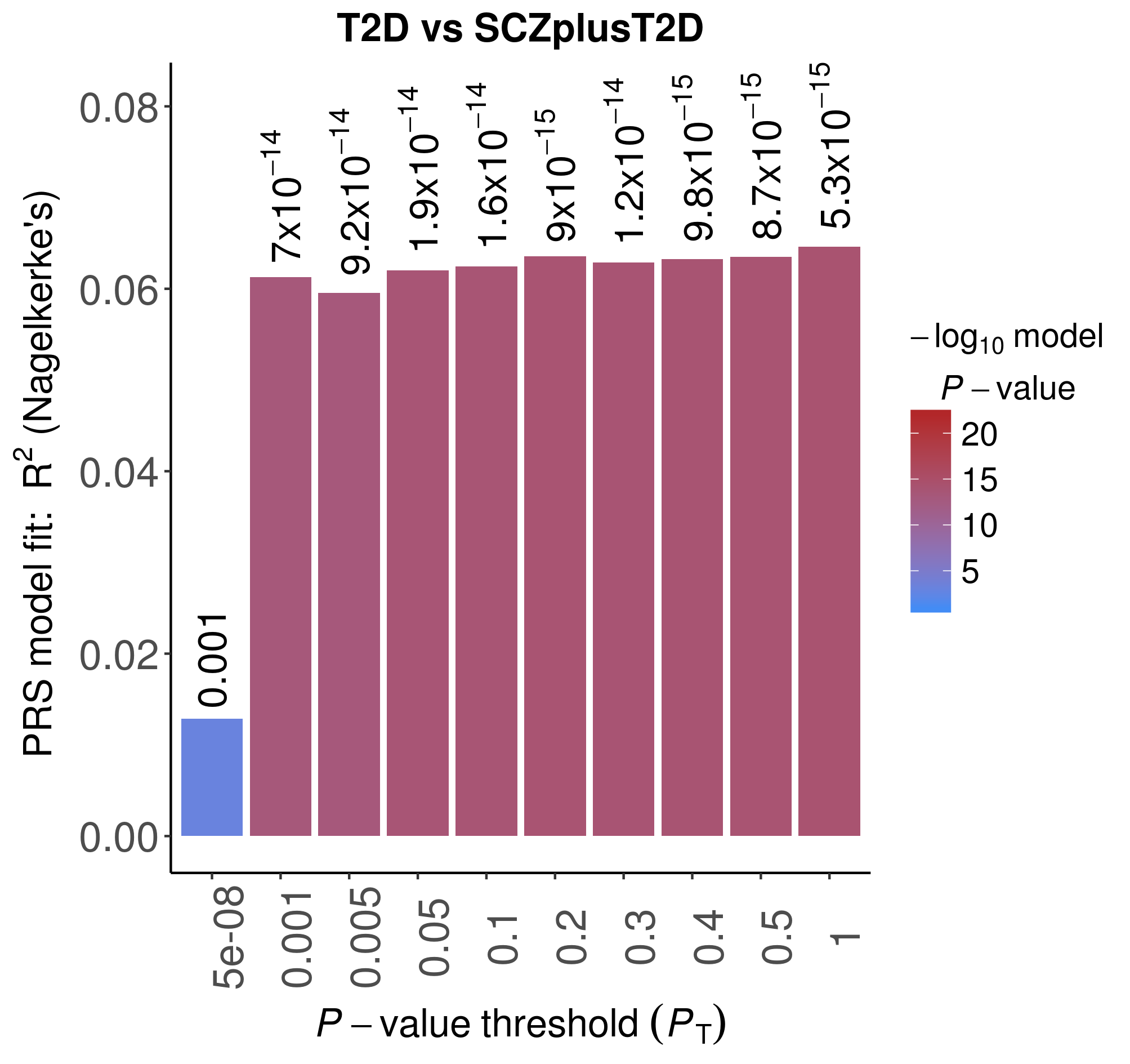
**

**
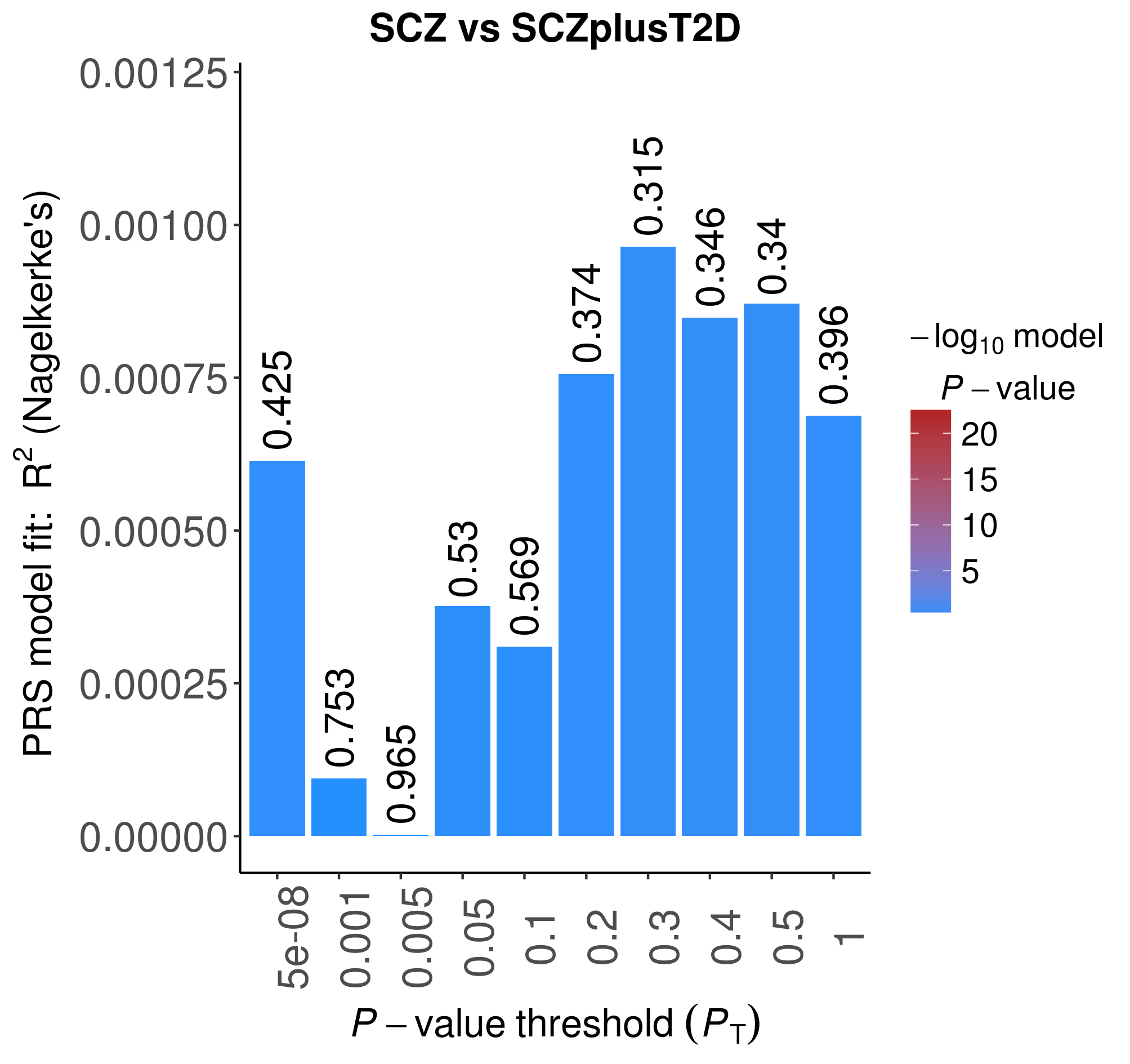

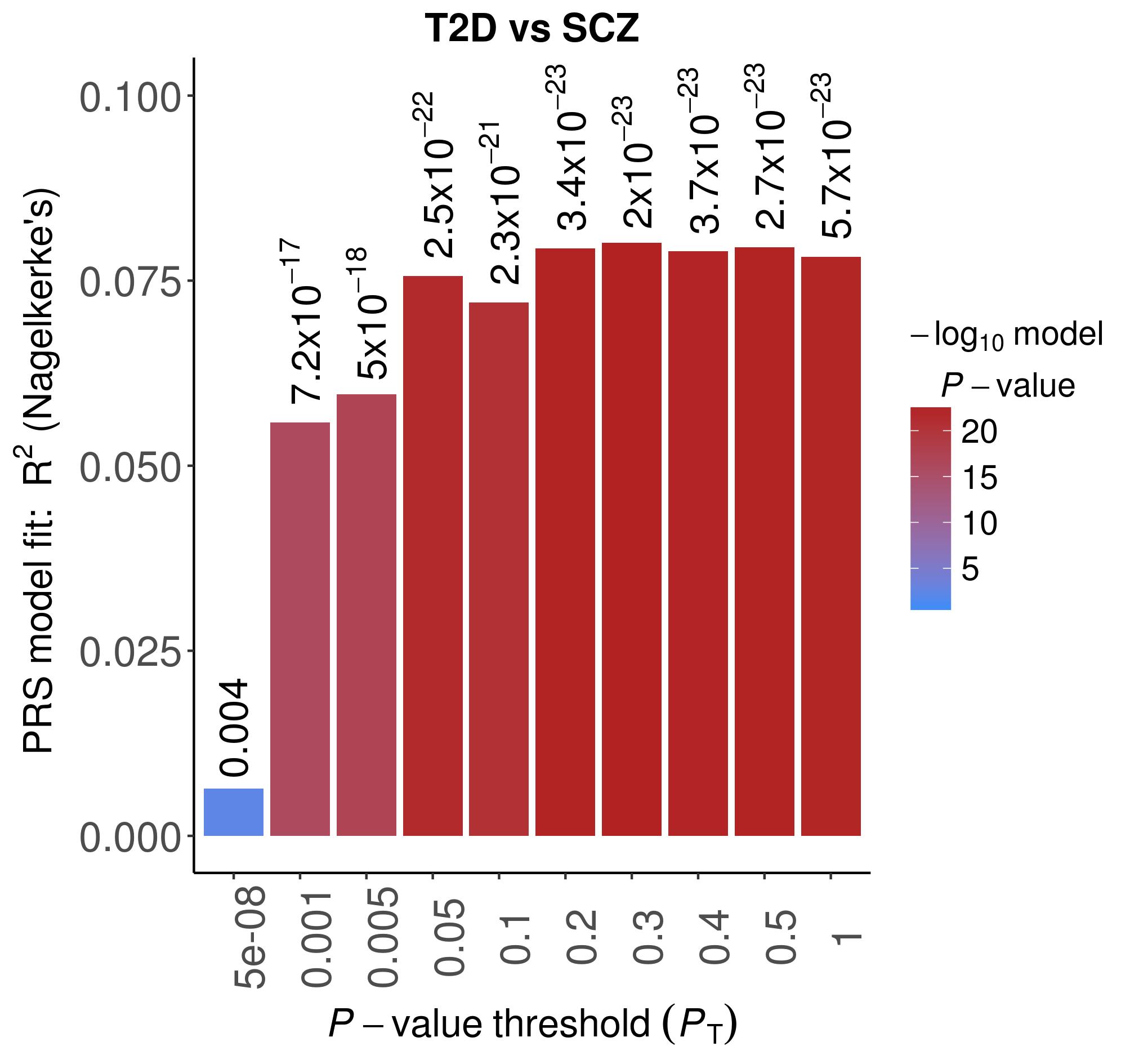
**

Nagelkerke's pseudo-R2 estimates are plotted at ten cumulative p-value thresholds. Note that the y-axis scales differ between plots.

**Supplementary Figure 5. Polygenic risk score analyses for T2D in GOMAP**

**
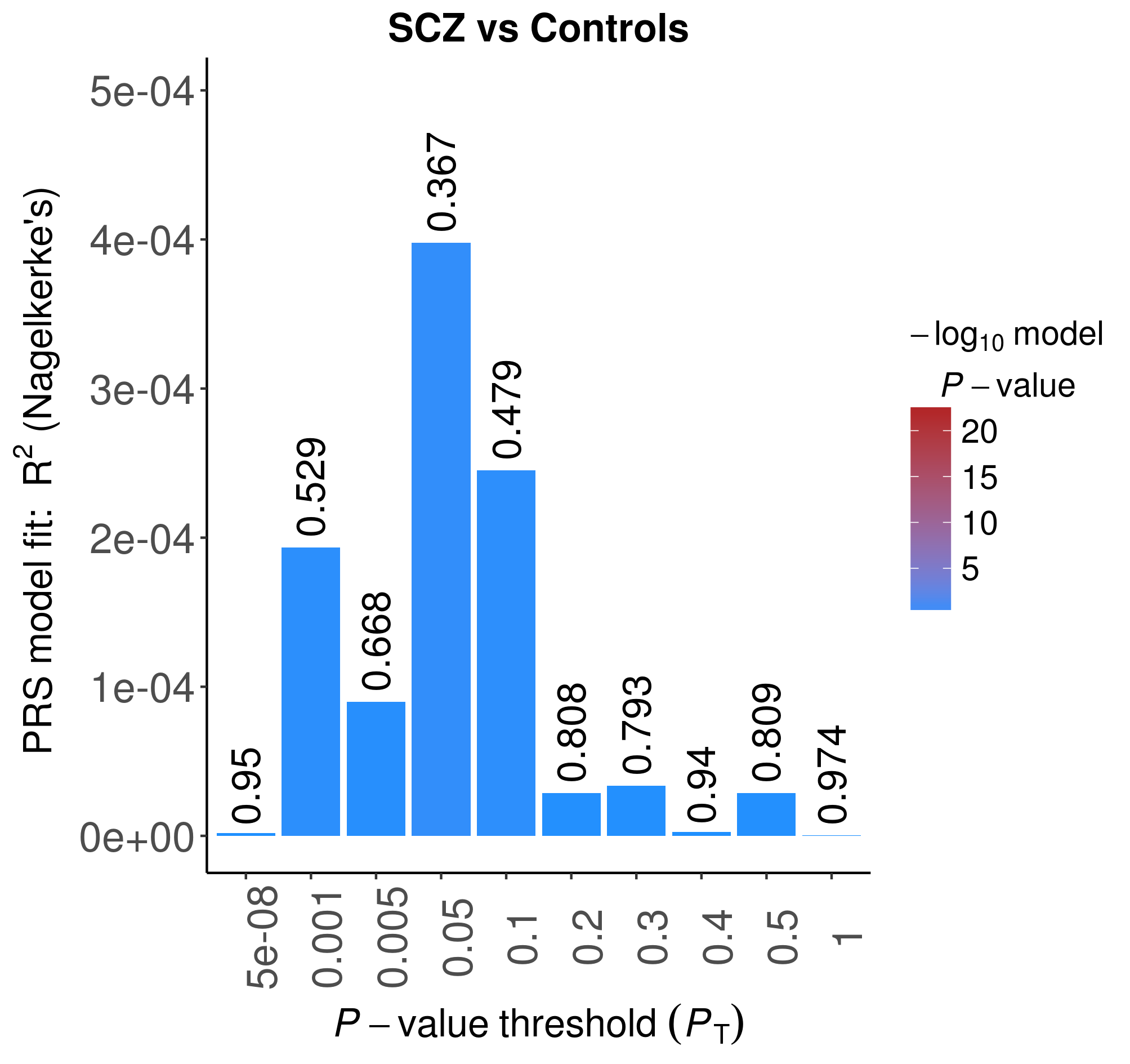

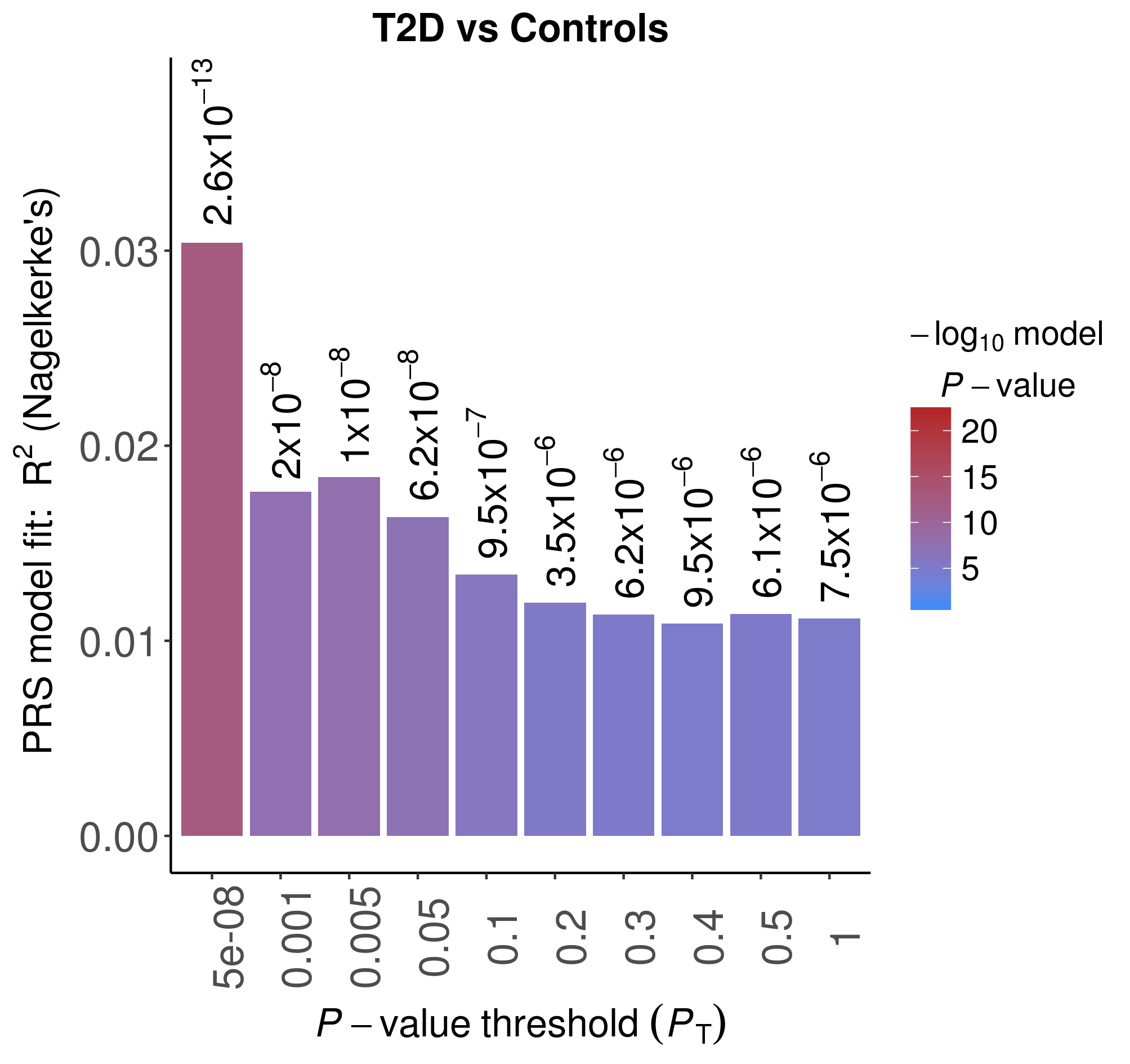
**

**
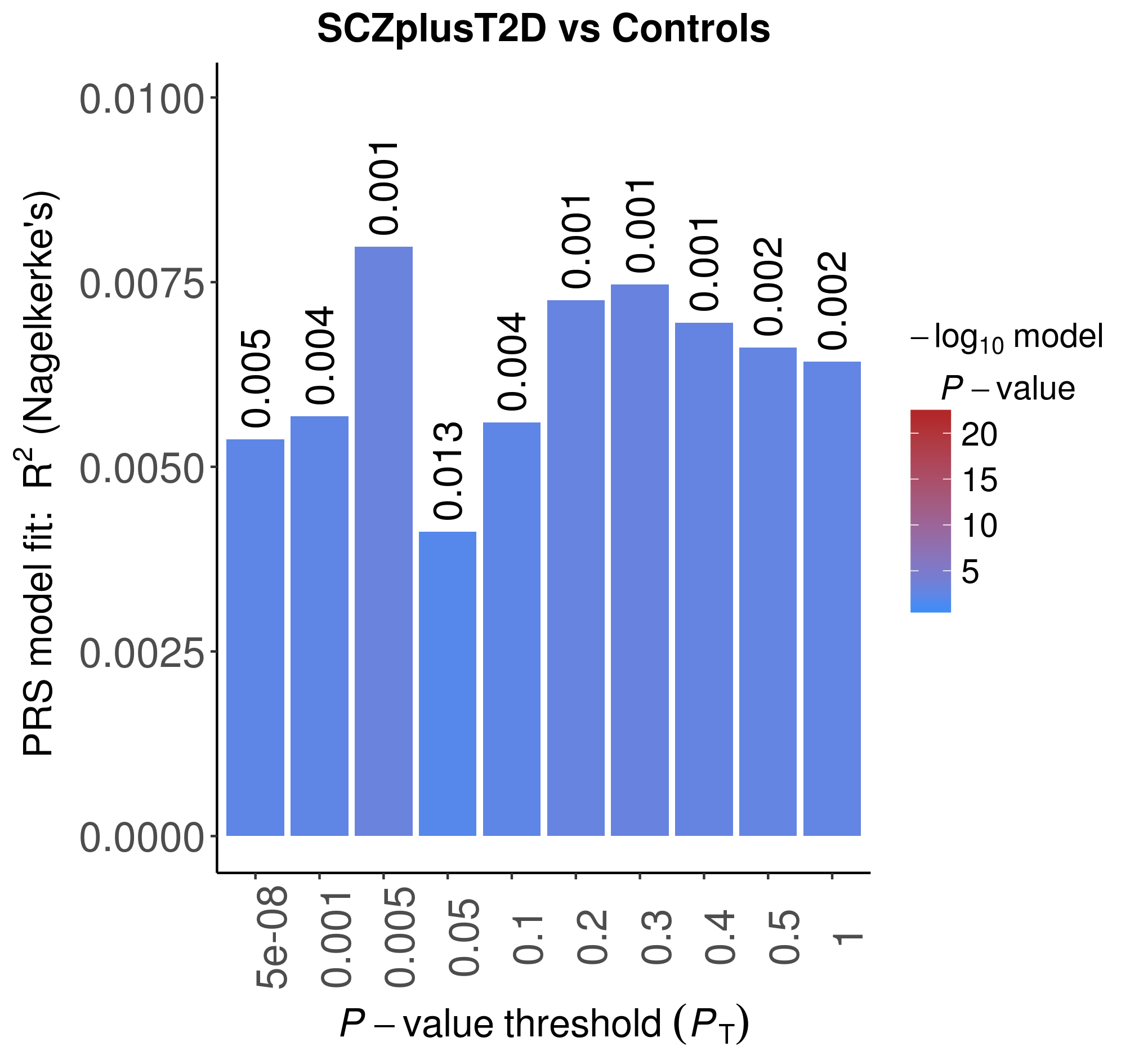

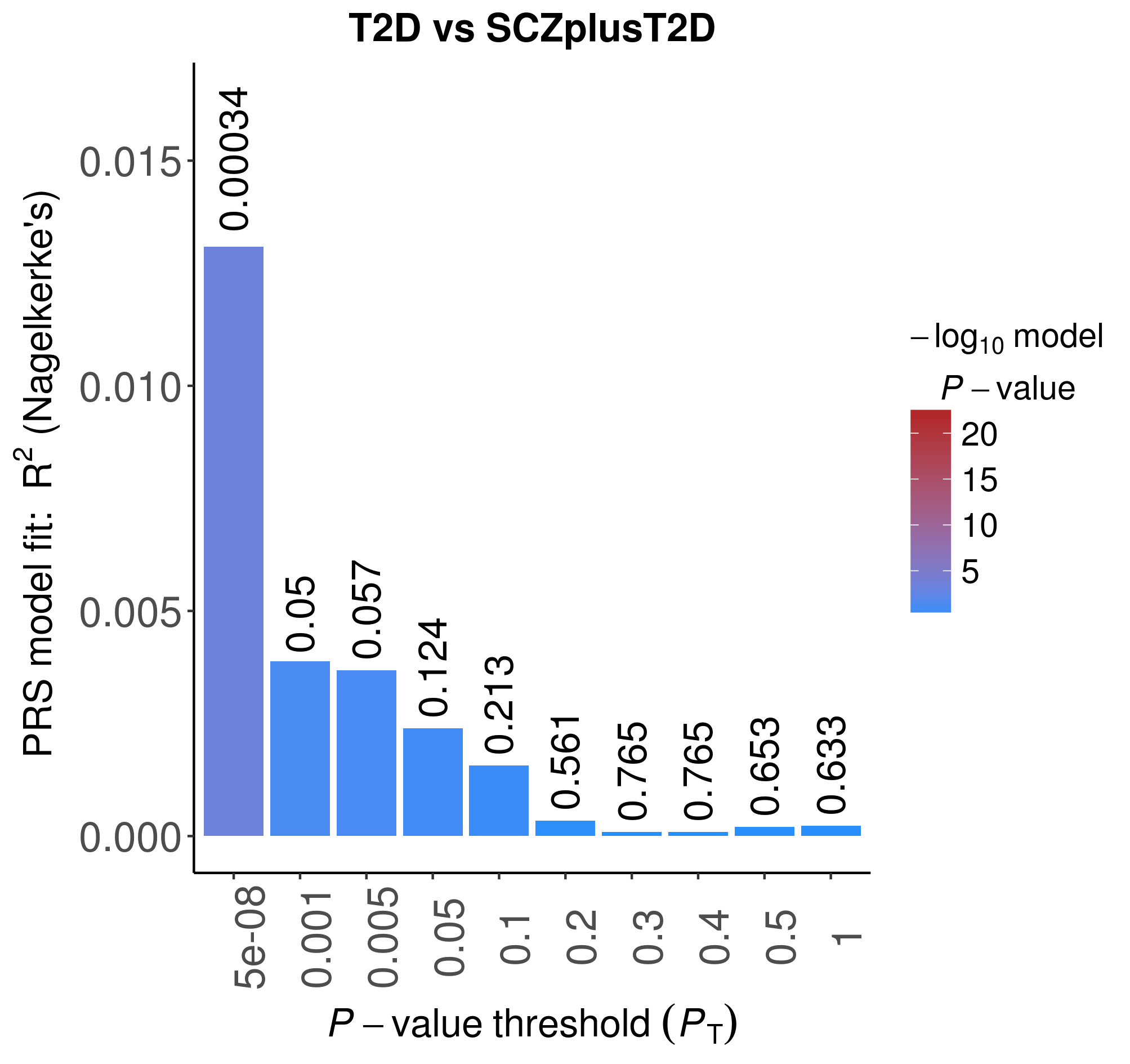
**

**
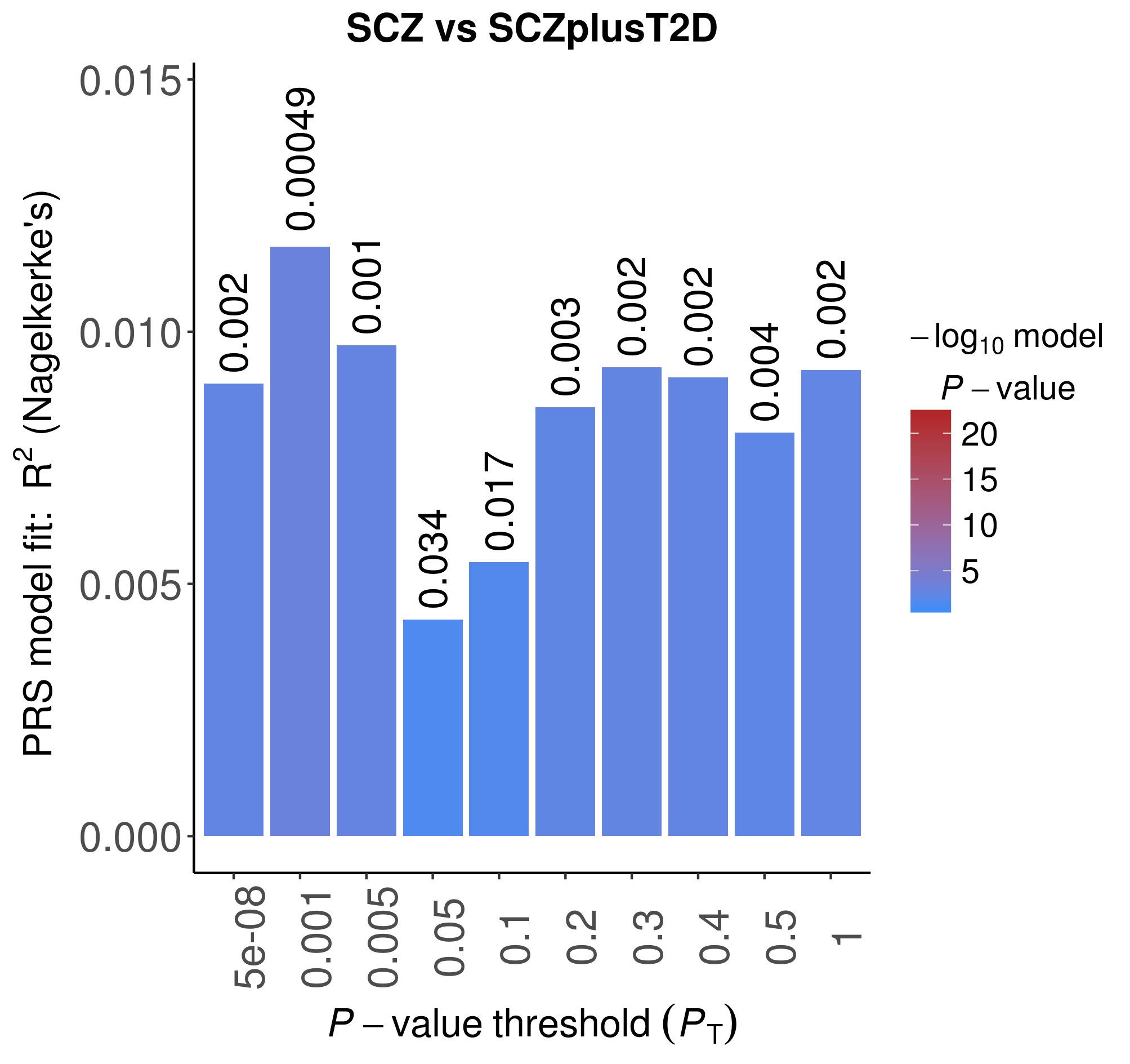

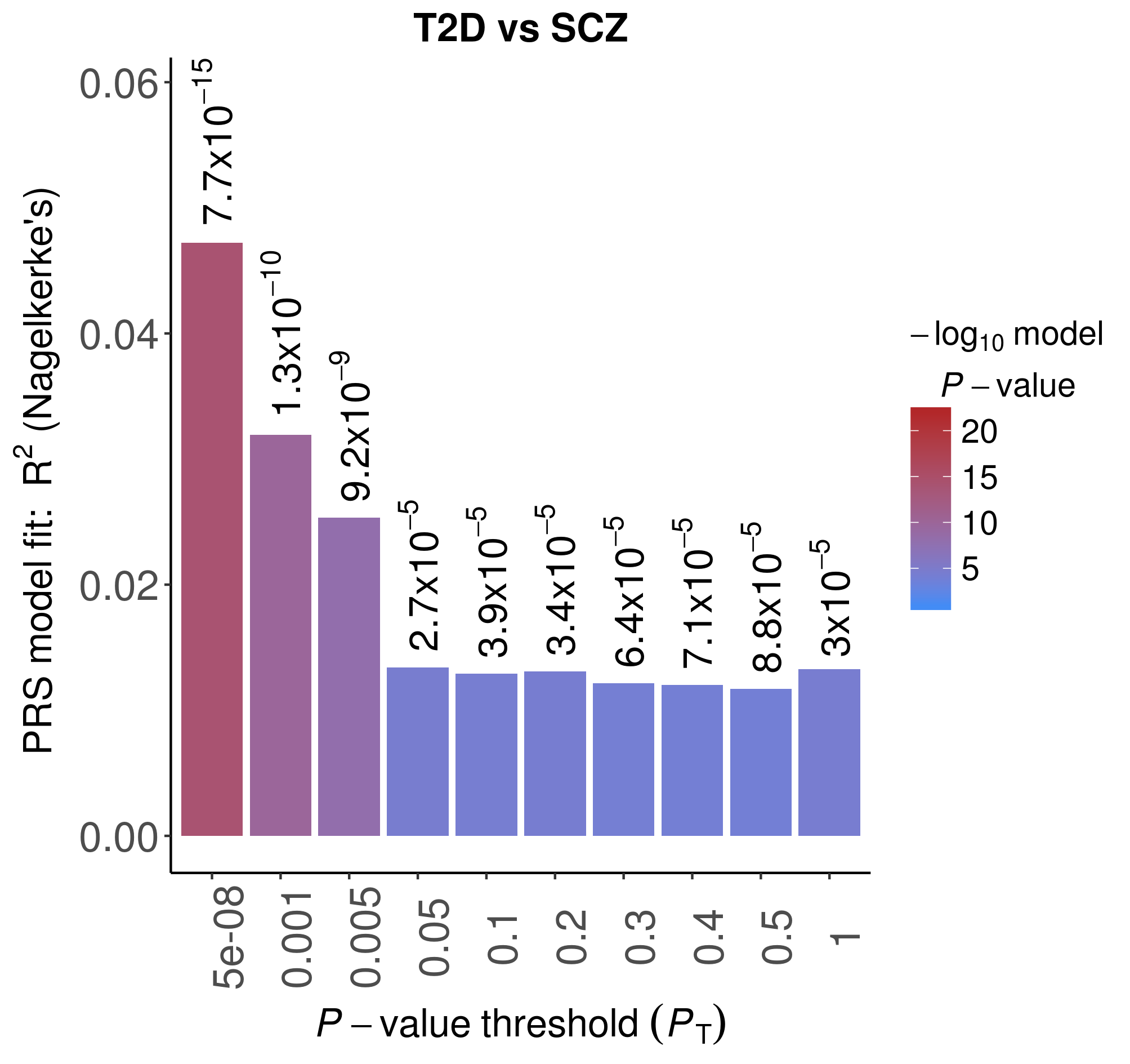
**

Nagelkerke's pseudo-R2 estimates are plotted at ten cumulative p-value thresholds. Note that the y-axis scales differ between plots.

**Supplementary Figure 6. Manhattan plots of case-control GWAS in GOMAP**

a)

**
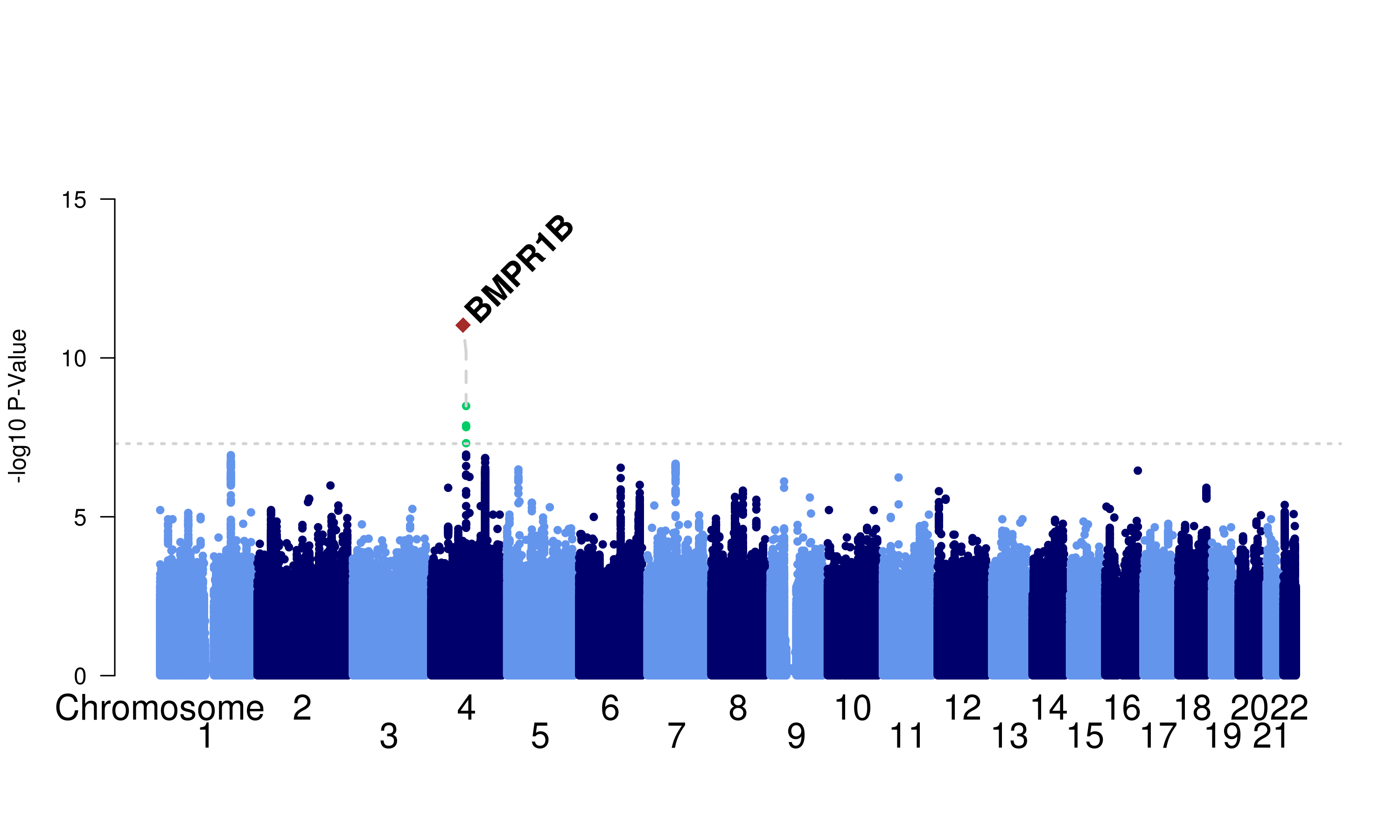
**

b)

**
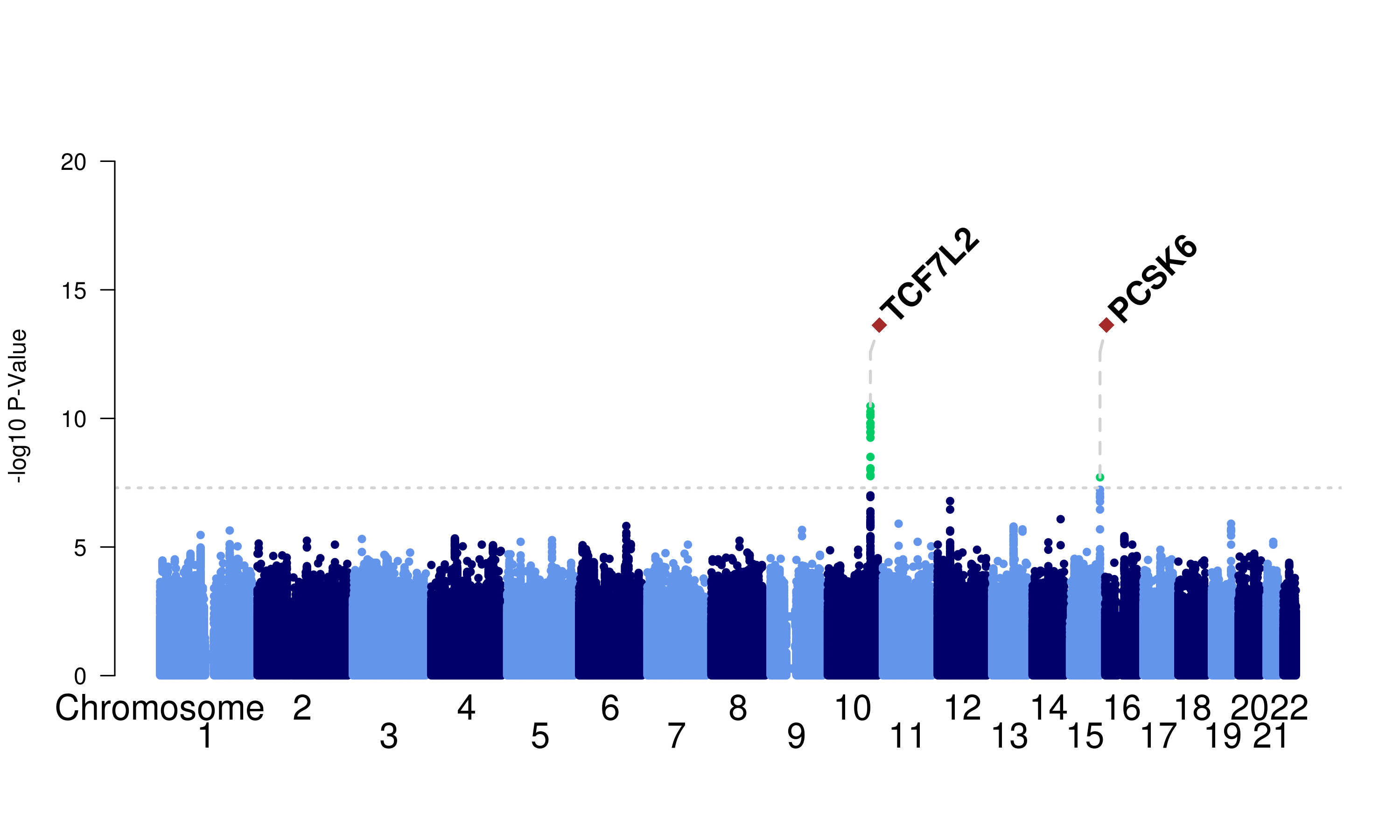
**

c)


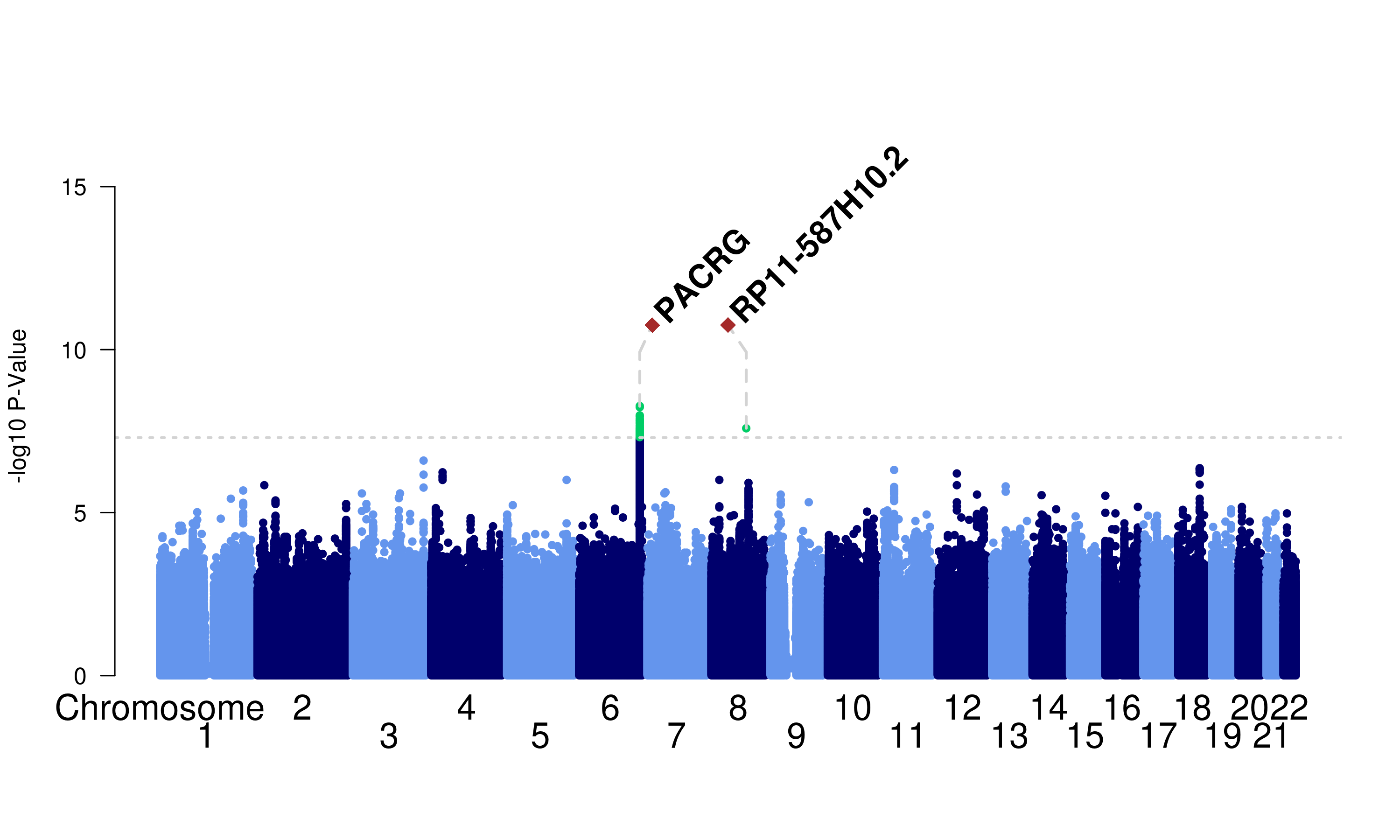


For each SNP the -log10 of its p-value is plotted against its chromosomal location. a) SCZ vs controls, b) T2D vs controls, c) SCZplusT2D vs controls

**Supplementary Figure 7. Manhattan plots of case-case GWAS in GOMAP**

**
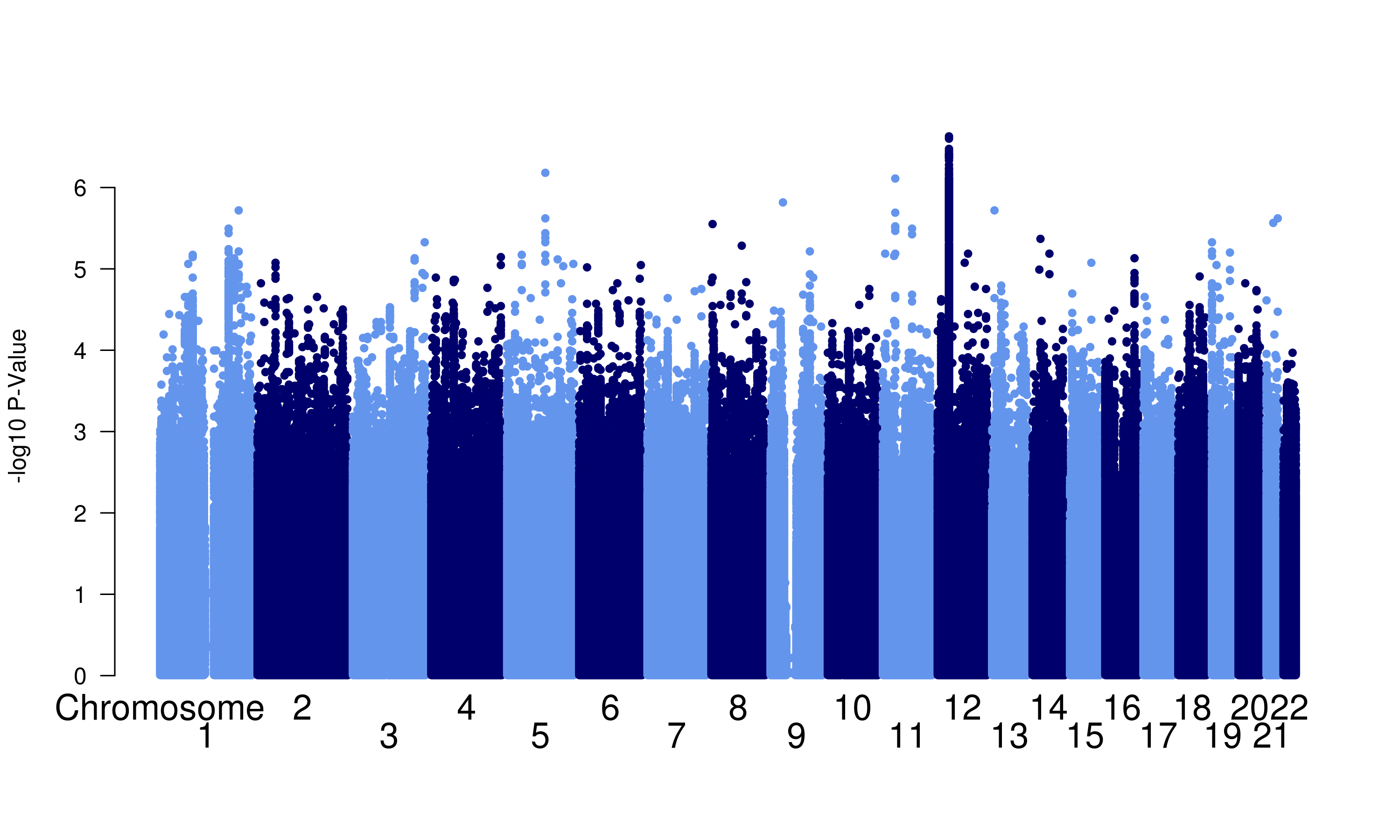
**

a)

b)

**
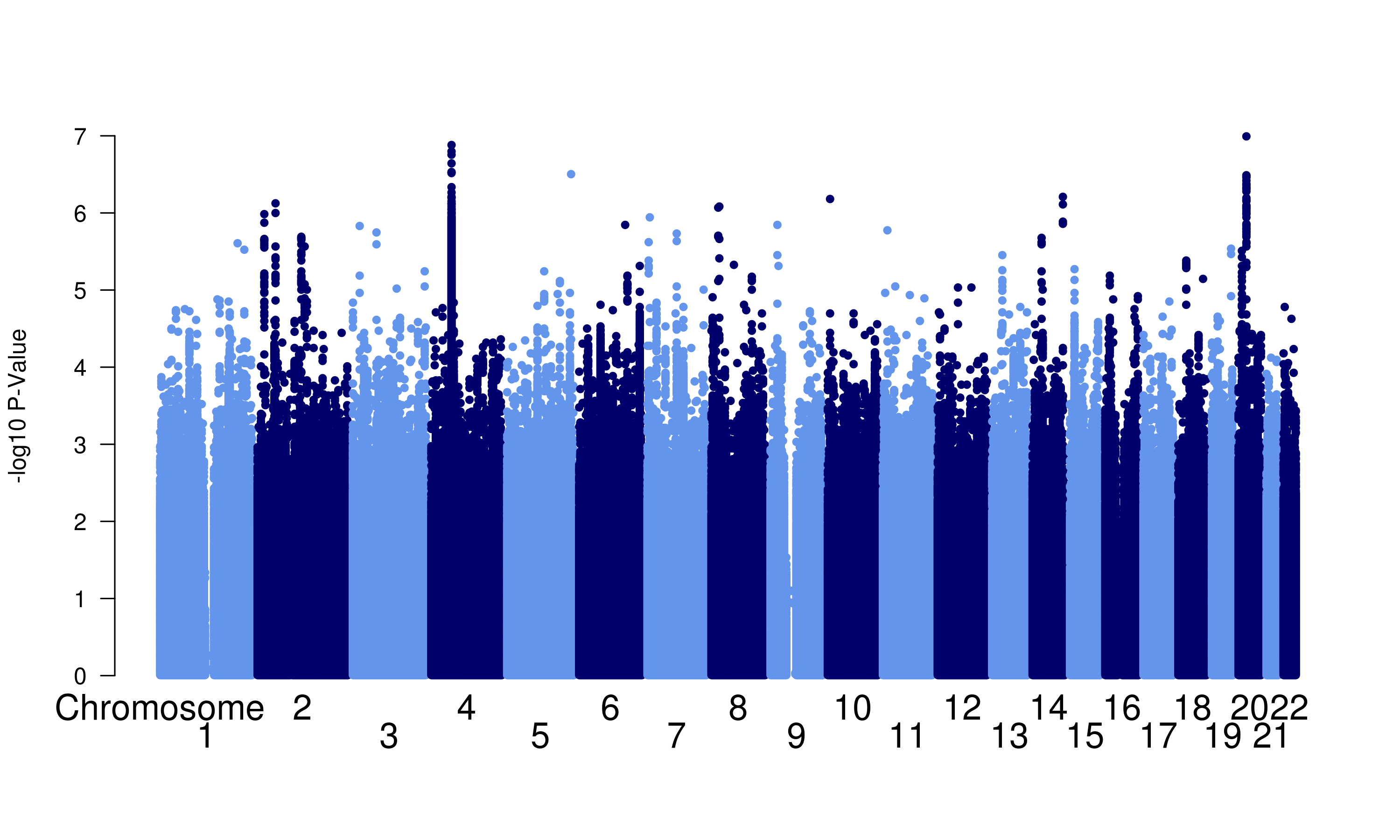
**

c)


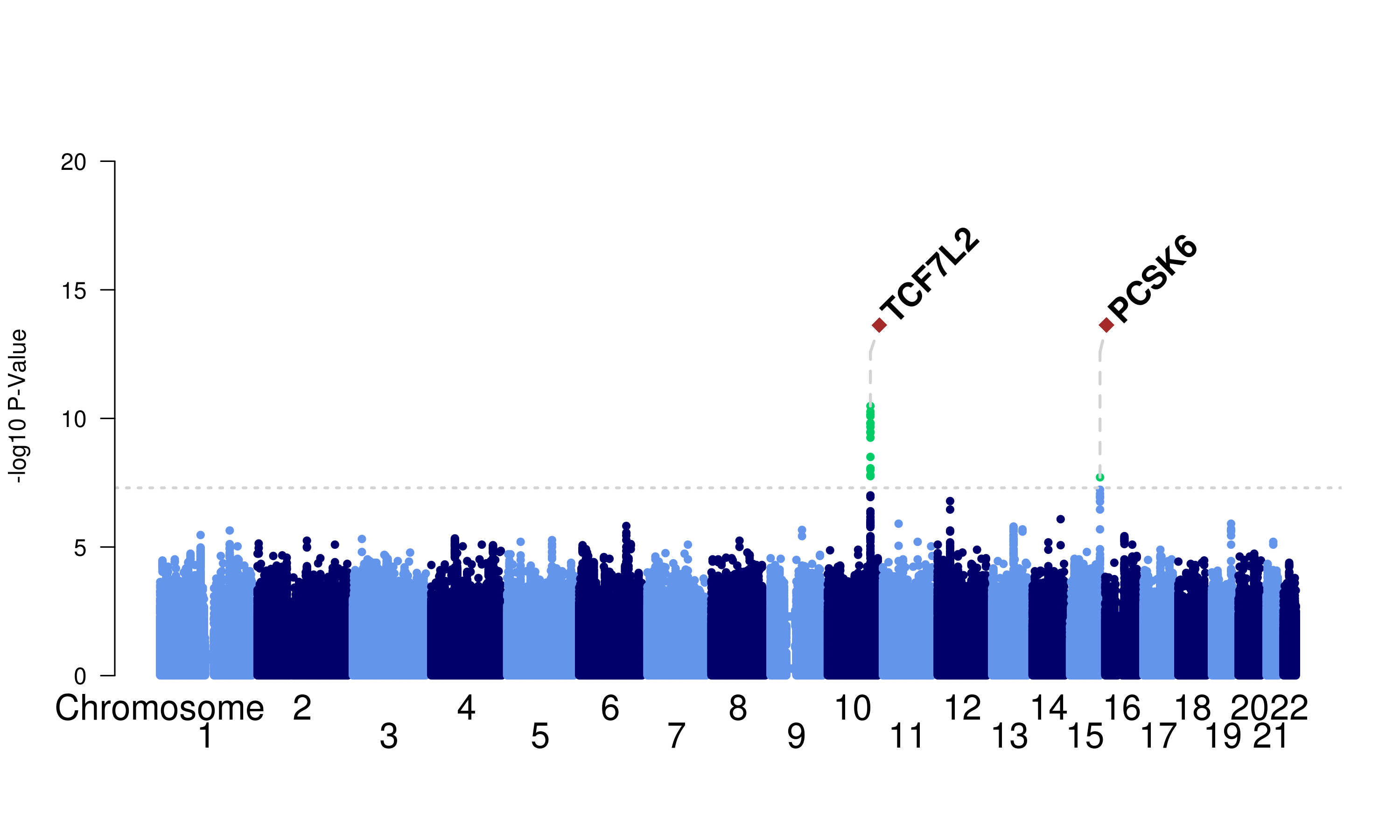


For each SNP the -log10 of its p-value is plotted against its chromosomal location. a) SCZ vs SCZplusT2D, b) T2D vs SCZplusT2D, c) SCZ vs T2D

**Supplementary Figure 8.** Quantile-quantile plots of case-control GWAS in GOMAP

b)

a)


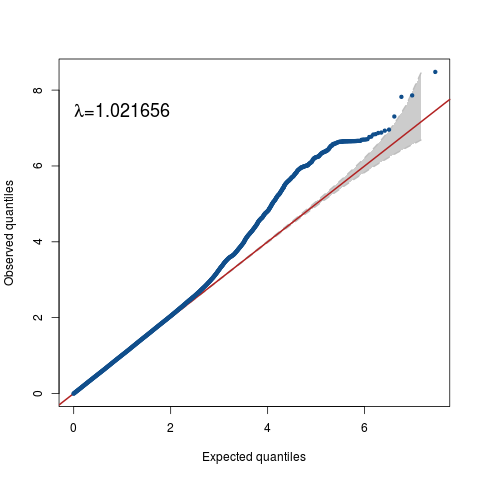

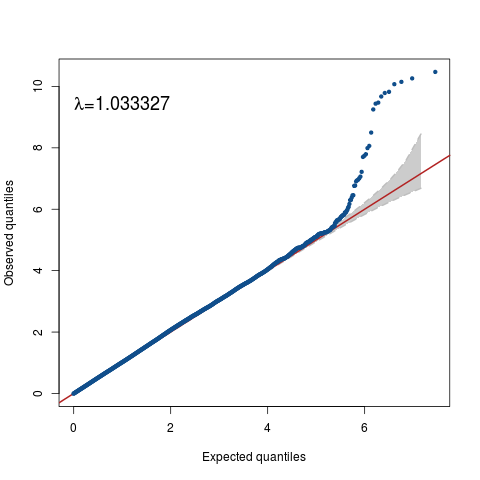


c)


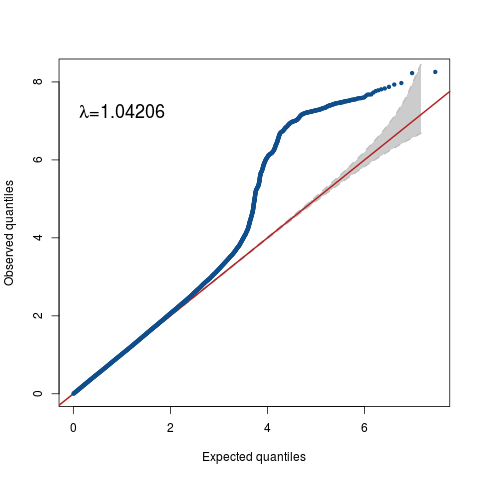


For each SNP the observed -log10 p-value is plotted against its expected value. a) SCZ vs controls, b) T2D vs controls, c) SCZplusT2D vs controls

**Supplementary Figure 9.** Quantile-quantile plots of case-case GWAS in GOMAP

b)

a)

c)

**
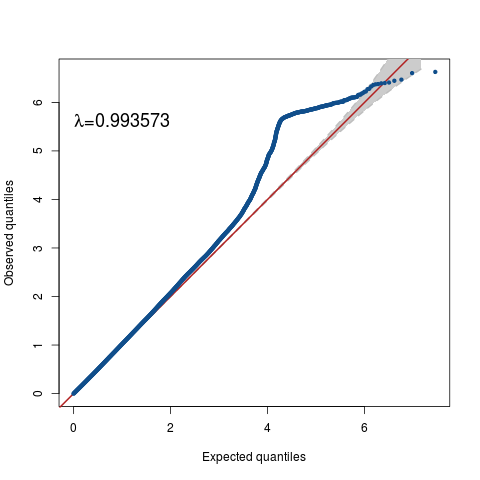

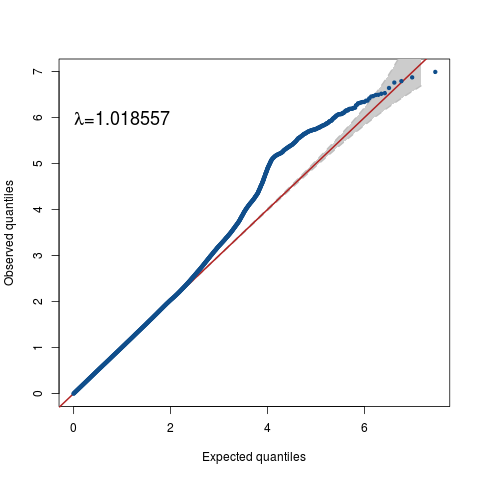
**

**
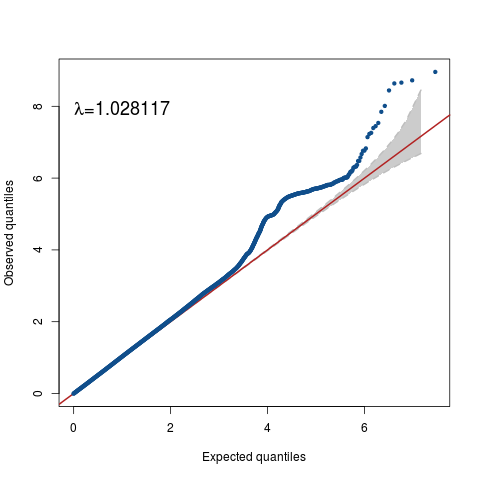
**

For each SNP the observed -log10 p-value is plotted against its expected value. a) SCZ vs SCZplusT2D, b) T2D vs SCZplusT2D, c) SCZ vs T2D

**Supplementary Table 1.** Number of individuals excluded during QC in GOMAP. The total number of samples refers to the SCZ, T2D and SCZplusT2D patient groups (excluding patients with psychiatric diagnoses other than SCZ, as these were not used in the analyses).

|  |  |
| --- | --- |
| **Total samples before QC** | **2,405** |
| QC step | Exclusions |
| Call rate < 90% | 5 |
| Sex mismatch | 42 |
| Heterozygosity outliers (± 3 SD), MAF ≥1% | 30 |
| Heterozygosity outliers (± 3SD), MAF <1% | 13 |
| Related and duplicated samples | 56 |
| Sample ID mismatch | 15 |
| Ethnic outliers | 7 |
| Total unique exclusions | 154 |
| **Total samples left** | **2,251** |

**Supplementary Table 2.** Number of variants excluded during QC in GOMAP

| **Total variants before QC** | **538,403** |
| --- | --- |
| QC step | Exclusions |
| Non-autosomal, non-chrX nonPAR | 2,512 |
| Call rate < 98% | 8,949 |
| HWE deviation p < 1x10-4 | 829 |
| cluster separation score < 0.4 | 1,126 |
| chrX-nonPAR and heterozygous haploid | 716 |
| Total exclusions | 14,132 |
| **Total variants left** | **524,271** |

**Supplementary Table 3. Established risk variants for SCZ used for genetic risk score analysis**

| **Variant** | **Chr** | **Pos (hg18)** | **EA** | **NEA** | **OR (95% CI)** | **P** |
| --- | --- | --- | --- | --- | --- | --- |
| rs115329265 | 6 | 28712247 | A | G | 1.21 (1.18-1.25) | 3.86E-32 |
| rs11191419 | 10 | 104612335 | A | T | 0.91 (0.88-0.93) | 9.24E-18 |
| rs2007044 | 12 | 2344960 | A | G | 0.91 (0.89-0.93) | 2.63E-17 |
| rs1702294 | 1 | 98501984 | T | C | 0.89 (0.86-0.92) | 2.79E-17 |
| chr2_200825237_I | 2 | 200825237 | AT | A | 0.91 (0.88-0.93) | 1.78E-14 |
| rs2851447 | 12 | 123665113 | C | G | 0.91 (0.89-0.94) | 2.19E-14 |
| chr7_2025096_I | 7 | 2025096 | A | ACT | 0.92 (0.90-0.94) | 6.12E-14 |
| chr10_104957618_I | 10 | 104957618 | CA | C | 0.84 (0.80-0.89) | 1.04E-13 |
| rs12887734 | 14 | 104046834 | T | G | 1.09 (1.07-1.11) | 1.17E-13 |
| rs4391122 | 5 | 60598543 | A | G | 0.92 (0.90-0.95) | 1.73E-13 |
| rs4129585 | 8 | 143312933 | A | C | 1.08 (1.06-1.10) | 2.03E-13 |
| rs13240464 | 7 | 110898915 | T | C | 1.08 (1.06-1.11) | 6.16E-13 |
| rs9636107 | 18 | 53200117 | A | G | 0.93 (0.91-0.95) | 9.09E-13 |
| rs35518360 | 4 | 103146890 | A | T | 0.87 (0.83-0.90) | 9.56E-13 |
| rs8042374 | 15 | 78908032 | A | G | 1.09 (1.07-1.12) | 1.87E-12 |
| rs4702 | 15 | 91426560 | A | G | 0.92 (0.90-0.95) | 2.30E-12 |
| rs11682175 | 2 | 57987593 | T | C | 0.93 (0.91-0.95) | 2.54E-12 |
| rs10791097 | 11 | 130718630 | T | G | 1.08 (1.06-1.10) | 2.88E-12 |
| rs6704768 | 2 | 233592501 | A | G | 0.93 (0.91-0.95) | 3.15E-12 |
| rs75968099 | 3 | 36858583 | T | C | 1.08 (1.06-1.10) | 3.39E-12 |
| rs72934570 | 18 | 53533189 | T | C | 0.87 (0.82-0.91) | 3.67E-12 |
| rs55661361 | 11 | 124613957 | A | G | 0.92 (0.90-0.95) | 3.68E-12 |
| rs12826178 | 12 | 57622371 | T | G | 0.85 (0.80-0.89) | 5.30E-12 |
| rs9607782 | 22 | 41587556 | A | T | 1.09 (1.07-1.12) | 6.76E-12 |
| rs11693094 | 2 | 185601420 | T | C | 0.93 (0.91-0.95) | 7.13E-12 |
| rs75059851 | 11 | 133822569 | A | G | 1.10 (1.07-1.12) | 1.23E-11 |
| rs6434928 | 2 | 198304577 | A | G | 0.93 (0.90-0.95) | 1.48E-11 |
| chr18_52749216_D | 18 | 52749216 | I2 | D | 1.08 (1.05-1.10) | 1.75E-11 |
| chr11_46350213_D | 11 | 46350213 | I2 | D | 0.90 (0.88-0.93) | 1.97E-11 |
| chr22_39987017_D | 22 | 39987017 | TA | T | 0.93 (0.91-0.95) | 2.20E-11 |
| rs7893279 | 10 | 18745105 | T | G | 1.12 (1.09-1.15) | 3.56E-11 |
| rs2535627 | 3 | 52845105 | T | C | 1.07 (1.05-1.09) | 3.96E-11 |
| rs17194490 | 3 | 2547786 | T | G | 1.10 (1.07-1.13) | 4.87E-11 |
| rs7432375 | 3 | 136288405 | A | G | 0.93 (0.91-0.95) | 5.27E-11 |
| chr3_180594593_I | 3 | 180594593 | TA | T | 0.91 (0.89-0.94) | 5.35E-11 |
| rs6065094 | 20 | 37453194 | A | G | 0.93 (0.91-0.95) | 5.52E-11 |
| rs7907645 | 10 | 104423800 | T | G | 1.14 (1.10-1.18) | 5.82E-11 |
| rs950169 | 15 | 84706461 | T | C | 0.92 (0.90-0.95) | 7.62E-11 |
| rs12704290 | 7 | 86427626 | A | G | 0.90 (0.87-0.93) | 1.04E-10 |
| rs36068923 | 8 | 111485761 | A | G | 0.92 (0.89-0.94) | 1.05E-10 |
| rs12691307 | 16 | 29939877 | A | G | 1.07 (1.05-1.09) | 1.30E-10 |
| rs12129573 | 1 | 73768366 | A | C | 1.07 (1.05-1.09) | 2.35E-10 |
| rs7405404 | 16 | 13749859 | T | C | 1.08 (1.06-1.11) | 3.93E-10 |
| rs2514218 | 11 | 113392994 | T | C | 0.93 (0.91-0.95) | 4.09E-10 |
| rs11210892 | 1 | 44100084 | A | G | 0.93 (0.91-0.95) | 4.97E-10 |
| rs4766428 | 12 | 110723245 | T | C | 1.07 (1.05-1.09) | 7.09E-10 |
| chr6_84280274_D | 6 | 84280274 | GC | G | 1.07 (1.05-1.09) | 8.57E-10 |
| rs140505938 | 1 | 150031490 | T | C | 0.91 (0.88-0.94) | 9.34E-10 |
| rs2973155 | 5 | 152608619 | T | C | 0.93 (0.91-0.96) | 1.02E-09 |
| rs12903146 | 15 | 61854663 | A | G | 1.07 (1.05-1.09) | 1.04E-09 |
| rs4523957 | 17 | 2208899 | T | G | 1.07 (1.05-1.09) | 1.04E-09 |
| rs1498232 | 1 | 30433951 | T | C | 1.07 (1.05-1.09) | 1.28E-09 |
| rs111294930 | 5 | 152177121 | A | G | 1.09 (1.06-1.12) | 1.31E-09 |
| rs6002655 | 22 | 42603814 | T | C | 1.07 (1.05-1.09) | 1.48E-09 |
| rs2332700 | 14 | 72417326 | C | G | 1.08 (1.05-1.10) | 1.69E-09 |
| rs6984242 | 8 | 60700469 | A | G | 0.94 (0.92-0.96) | 1.76E-09 |
| rs77502336 | 11 | 123394636 | C | G | 1.07 (1.05-1.09) | 2.01E-09 |
| chr1_8424984_D | 1 | 8424984 | GA | G | 1.07 (1.05-1.09) | 2.03E-09 |
| rs6466055 | 7 | 104929064 | A | C | 1.07 (1.05-1.09) | 2.46E-09 |
| rs11139497 | 9 | 84739941 | A | T | 1.07 (1.05-1.09) | 3.09E-09 |
| rs11027857 | 11 | 24403620 | A | G | 1.06 (1.04-1.09) | 3.21E-09 |
| rs2053079 | 19 | 30987423 | A | G | 0.93 (0.91-0.95) | 3.79E-09 |
| rs4648845 | 1 | 2387101 | T | C | 1.07 (1.05-1.09) | 4.03E-09 |
| rs77149735 | 1 | 243555105 | A | G | 1.33 (1.23-1.42) | 4.40E-09 |
| rs3849046 | 5 | 137851192 | T | C | 1.06 (1.04-1.09) | 4.83E-09 |
| rs2239063 | 12 | 2511831 | A | C | 1.07 (1.05-1.09) | 5.39E-09 |
| rs9922678 | 16 | 9946319 | A | G | 1.07 (1.05-1.09) | 6.72E-09 |
| rs8082590 | 17 | 17958402 | A | G | 0.94 (0.91-0.96) | 6.84E-09 |
| rs2905426 | 19 | 19478022 | T | G | 0.94 (0.92-0.96) | 6.92E-09 |
| rs3735025 | 7 | 137074844 | T | C | 1.07 (1.04-1.09) | 7.75E-09 |
| rs75575209 | 2 | 58138192 | A | T | 0.90 (0.86-0.93) | 1.01E-08 |
| rs10520163 | 4 | 170626552 | T | C | 1.06 (1.04-1.08) | 1.02E-08 |
| chr2_146436222_I | 2 | 146436222 | TC | T | 1.08 (1.06-1.11) | 1.07E-08 |
| rs59979824 | 2 | 193848340 | A | C | 0.94 (0.91-0.96) | 1.08E-08 |
| rs78322266 | 18 | 53063676 | T | G | 1.19 (1.13-1.25) | 1.10E-08 |
| rs11685299 | 2 | 225391296 | A | C | 0.94 (0.92-0.96) | 1.11E-08 |
| rs1106568 | 4 | 176861301 | A | G | 0.93 (0.91-0.96) | 1.15E-08 |
| rs12325245 | 16 | 58681393 | A | T | 0.92 (0.89-0.95) | 1.15E-08 |
| rs215411 | 4 | 23423603 | A | T | 1.07 (1.04-1.09) | 1.22E-08 |
| rs1501357 | 5 | 45364875 | T | C | 0.93 (0.90-0.95) | 1.24E-08 |
| rs16867576 | 5 | 88746331 | A | G | 1.10 (1.07-1.13) | 1.36E-08 |
| rs2693698 | 14 | 99719219 | A | G | 0.94 (0.92-0.96) | 1.38E-08 |
| rs55833108 | 10 | 104741583 | T | G | 1.08 (1.05-1.10) | 1.42E-08 |
| rs9841616 | 3 | 181167585 | A | T | 0.92 (0.89-0.95) | 1.65E-08 |
| rs117074560 | 6 | 96459651 | T | C | 0.86 (0.80-0.91) | 1.66E-08 |
| rs10803138 | 1 | 243555219 | A | G | 0.93 (0.91-0.96) | 1.79E-08 |
| rs7819570 | 8 | 89588626 | T | G | 1.08 (1.05-1.11) | 1.90E-08 |
| rs73229090 | 8 | 27442127 | A | C | 0.91 (0.87-0.94) | 1.95E-08 |
| rs10043984 | 5 | 137712121 | T | C | 1.07 (1.05-1.09) | 2.18E-08 |
| rs12522290 | 5 | 152797656 | C | G | 1.09 (1.06-1.11) | 2.23E-08 |
| rs7801375 | 7 | 131567263 | A | G | 0.92 (0.89-0.95) | 2.26E-08 |
| rs832187 | 3 | 63833050 | T | C | 0.94 (0.92-0.96) | 2.58E-08 |
| chr2_149429178_D | 2 | 149429178 | AT | A | 0.86 (0.80-0.91) | 2.62E-08 |
| rs10503253 | 8 | 4180844 | A | C | 1.07 (1.05-1.10) | 2.69E-08 |
| chr1_243881945_I | 1 | 243881945 | AT | A | 1.07 (1.04-1.09) | 3.11E-08 |
| rs8044995 | 16 | 68189340 | A | G | 1.08 (1.05-1.11) | 3.27E-08 |
| rs6704641 | 2 | 200164252 | A | G | 1.08 (1.05-1.11) | 3.40E-08 |
| rs715170 | 18 | 53795514 | T | C | 0.94 (0.91-0.96) | 3.47E-08 |
| rs79212538 | 5 | 151993104 | T | G | 1.15 (1.10-1.20) | 3.84E-08 |
| rs11740474 | 5 | 153680747 | A | T | 0.94 (0.92-0.96) | 3.94E-08 |
| rs2068012 | 14 | 30190316 | T | C | 0.93 (0.91-0.96) | 4.14E-08 |
| rs2909457 | 2 | 162845855 | A | G | 0.94 (0.92-0.96) | 4.38E-08 |
| rs56205728 | 15 | 40567237 | A | G | 1.07 (1.05-1.09) | 4.92E-08 |
| rs1023500 | 22 | 42340844 | T | C | 1.08 (1.05-1.10) | 5.04E-08 |
| rs12148337 | 15 | 70589272 | T | C | 1.06 (1.04-1.08) | 5.33E-08 |
| rs4330281 | 3 | 17859366 | T | C | 0.94 (0.92-0.96) | 5.51E-08 |
| rs9420 | 11 | 57510294 | A | G | 1.06 (1.04-1.09) | 6.65E-08 |
| rs1339227 | 6 | 73155701 | T | C | 0.94 (0.92-0.96) | 6.86E-08 |
| rs679087 | 12 | 29917265 | A | C | 0.94 (0.92-0.96) | 7.06E-08 |
| rs190065944 | 15 | 78859610 | A | G | 1.08 (1.05-1.11) | 7.22E-08 |
| rs10860964 | 12 | 103596455 | T | C | 1.06 (1.04-1.08) | 9.92E-08 |
| rs4388249 | 5 | 109036066 | T | C | 1.07 (1.05-1.10) | 1.03E-07 |
| rs4240748 | 12 | 92246786 | C | G | 0.94 (0.92-0.96) | 1.03E-07 |
| rs6670165 | 1 | 177280121 | T | C | 1.07 (1.05-1.10) | 1.16E-07 |
| rs7267348 | 20 | 48131036 | T | C | 0.94 (0.91-0.96) | 1.18E-07 |
| rs3768644 | 2 | 72361505 | A | G | 0.91 (0.88-0.95) | 1.30E-07 |
| rs14403 | 1 | 243663893 | T | C | 0.93 (0.91-0.96) | 1.31E-07 |
| rs76869799 | 1 | 97834525 | C | G | 0.85 (0.79-0.91) | 1.44E-07 |
| rs7523273 | 1 | 207977083 | A | G | 1.06 (1.04-1.08) | 1.61E-07 |
| rs12421382 | 11 | 109378071 | T | C | 0.94 (0.92-0.96) | 1.72E-07 |
| rs324017 | 12 | 57487814 | A | C | 0.94 (0.92-0.96) | 2.13E-07 |
| rs56873913 | 19 | 50091199 | T | G | 1.07 (1.04-1.09) | 2.19E-07 |
| chr7_24747494_D | 7 | 24747494 | C | CTA | 1.09 (1.06-1.13) | 3.59E-07 |
| chr5_140143664_I | 5 | 140143664 | CATTGAAAGAAA | C | 1.05 (1.03-1.08) | 3.60E-07 |
| rs211829 | 7 | 110048893 | T | C | 1.06 (1.04-1.08) | 5.47E-07 |

Shown are 125 autosomal variants associated with schizophrenia in Ref. 11 that were used to construct genetic risk scores in GOMAP. For each variant, chromosome position, effect (EA) and alternative (NEA) allele, as well as odds ratios with 95% confidence interval and p-values are given.

**Supplementary Table 4. Established risk variants for T2D used for genetic risk score analysis**

| **Variant** | **Chr** | **Pos (hg18)** | **EA** | **NEA** | **OR (95% CI)** | **P** |
| --- | --- | --- | --- | --- | --- | --- |
| rs7903146 | 10 | 114758349 | T | C | 1.40 (1.35-1.46) | 5.50E-65 |
| rs7756992 | 6 | 20679709 | G | A | 1.20 (1.16-1.25) | 1.30E-22 |
| rs1111875 | 10 | 94462882 | C | T | 1.15 (1.11-1.18) | 1.10E-15 |
| rs10811661 | 9 | 22134094 | T | C | 1.18 (1.13-1.23) | 1.50E-13 |
| rs3802177 | 8 | 118185025 | G | A | 1.16 (1.11-1.22) | 2.10E-11 |
| rs4402960 | 3 | 185511687 | T | G | 1.13 (1.09-1.17) | 2.70E-11 |
| rs9936385 | 16 | 53819169 | C | T | 1.13 (1.09-1.18) | 4.70E-11 |
| rs849135 | 7 | 28196413 | G | A | 1.12 (1.08-1.16) | 3.40E-10 |
| rs1801282 | 3 | 12393125 | C | G | 1.16 (1.11-1.22) | 5.00E-09 |
| rs13233731 | 7 | 130437689 | G | A | 1.10 (1.06-1.13) | 4.30E-08 |
| rs17791513 | 9 | 81905590 | A | G | 1.21 (1.13-1.30) | 1.00E-07 |
| rs2261181 | 12 | 66212318 | T | C | 1.16 (1.10-1.23) | 1.00E-07 |
| rs12571751 | 10 | 80942631 | A | G | 1.09 (1.06-1.13) | 1.80E-07 |
| rs4458523 | 4 | 6289986 | G | T | 1.09 (1.06-1.13) | 1.90E-07 |
| rs1552224 | 11 | 72433098 | A | C | 1.13 (1.08-1.19) | 4.90E-07 |
| rs17168486 | 7 | 14898282 | T | C | 1.13 (1.08-1.18) | 6.90E-07 |
| rs516946 | 8 | 41519248 | C | T | 1.10 (1.06-1.15) | 7.30E-07 |
| rs10830963 | 11 | 92708710 | G | C | 1.11 (1.07-1.16) | 7.30E-07 |
| rs1359790 | 13 | 80717156 | G | A | 1.10 (1.06-1.14) | 9.20E-07 |
| rs12427353 | 12 | 121426901 | G | C | 1.12 (1.07-1.17) | 1.00E-06 |
| rs6878122 | 5 | 76427311 | G | A | 1.13 (1.07-1.18) | 1.20E-06 |
| rs10203174 | 2 | 43690030 | C | T | 1.15 (1.08-1.21) | 1.50E-06 |
| rs7593730 | 2 | 161171454 | C | T | 1.11 (1.06-1.15) | 1.50E-06 |
| rs2943640 | 2 | 227093585 | C | A | 1.09 (1.05-1.12) | 1.80E-06 |
| rs4430796 | 17 | 36098040 | G | A | 1.13 (1.07-1.19) | 2.40E-06 |
| rs7955901 | 12 | 71433293 | C | T | 1.09 (1.05-1.13) | 3.20E-06 |
| rs5215 | 11 | 17408630 | C | T | 1.08 (1.05-1.12) | 4.40E-06 |
| rs9505118 | 6 | 7290437 | A | G | 1.08 (1.05-1.12) | 6.10E-06 |
| rs11634397 | 15 | 80432222 | G | A | 1.09 (1.05-1.13) | 7.30E-06 |
| rs11717195 | 3 | 123082398 | T | C | 1.09 (1.05-1.14) | 9.70E-06 |
| rs243088 | 2 | 60568745 | T | A | 1.09 (1.05-1.13) | 1.00E-05 |
| rs7845219 | 8 | 95937502 | T | C | 1.08 (1.04-1.12) | 1.40E-05 |
| rs702634 | 5 | 53271420 | A | G | 1.08 (1.04-1.12) | 1.80E-05 |
| rs163184 | 11 | 2847069 | G | T | 1.09 (1.05-1.13) | 1.90E-05 |
| rs3130501 | 6 | 31136453 | G | A | 1.09 (1.05-1.13) | 2.00E-05 |
| rs7202877 | 16 | 75247245 | T | G | 1.15 (1.08-1.22) | 2.30E-05 |
| rs12899811 | 15 | 91544076 | G | A | 1.09 (1.04-1.13) | 3.30E-05 |
| rs6813195 | 4 | 153520475 | C | T | 1.08 (1.04-1.12) | 6.10E-05 |
| rs2075423 | 1 | 214154719 | G | T | 1.08 (1.04-1.12) | 6.70E-05 |
| rs7178572 | 15 | 77747190 | G | A | 1.08 (1.04-1.12) | 1.00E-04 |
| rs12970134 | 18 | 57884750 | A | G | 1.08 (1.04-1.12) | 1.10E-04 |
| rs6808574 | 3 | 187740523 | C | T | 1.08 (1.04-1.12) | 1.30E-04 |
| rs11063069 | 12 | 4374373 | G | A | 1.10 (1.05-1.15) | 1.50E-04 |
| rs10842994 | 12 | 27965150 | C | T | 1.09 (1.04-1.13) | 1.50E-04 |
| rs6795735 | 3 | 64705365 | C | T | 1.07 (1.03-1.10) | 2.30E-04 |
| rs2796441 | 9 | 84308948 | G | A | 1.07 (1.03-1.12) | 2.50E-04 |
| rs10923931 | 1 | 120517959 | T | G | 1.10 (1.05-1.16) | 3.10E-04 |
| rs10401969 | 19 | 19407718 | C | T | 1.13 (1.05-1.21) | 5.40E-04 |
| rs2334499 | 11 | 1696849 | T | C | 1.07 (1.03-1.11) | 7.30E-04 |
| rs4275659 | 12 | 123447928 | C | T | 1.06 (1.03-1.10) | 8.80E-04 |
| rs7612463 | 3 | 23336450 | C | A | 1.10 (1.04-1.16) | 9.80E-04 |
| rs17106184 | 1 | 50909985 | G | A | 1.10 (1.04-1.17) | 1.10E-03 |
| rs7163757 | 15 | 62391608 | C | T | 1.06 (1.02-1.10) | 1.30E-03 |
| rs8108269 | 19 | 46158513 | G | T | 1.06 (1.02-1.11) | 3.10E-03 |
| rs4812829 | 20 | 42989267 | A | G | 1.07 (1.02-1.12) | 9.10E-03 |
| rs7041847 | 9 | 4287466 | A | G | 1.05 (1.01-1.09) | 9.90E-03 |
| rs11257655 | 10 | 12307894 | T | C | 1.06 (1.01-1.11) | 1.30E-02 |
| rs10278336 | 7 | 44245363 | A | G | 1.05 (1.01-1.09) | 2.00E-02 |
| rs459193 | 5 | 55806751 | G | A | 1.05 (1.01-1.09) | 2.10E-02 |
| rs780094 | 2 | 27741237 | C | T | 1.04 (1.00-1.08) | 2.50E-02 |
| rs3923113 | 2 | 165501849 | A | C | 1.04 (1.00-1.08) | 3.10E-02 |
| rs2028299 | 15 | 90374257 | C | A | 1.04 (1.00-1.09) | 3.50E-02 |
| rs831571 | 3 | 64048297 | C | T | 1.03 (0.99-1.08) | 1.60E-01 |
| rs1802295 | 10 | 70931474 | T | C | 1.02 (0.98-1.06) | 2.80E-01 |
| rs3786897 | 19 | 33893008 | A | G | 1.02 (0.98-1.06) | 3.10E-01 |
| rs7403531 | 15 | 38822905 | T | C | 1.02 (0.98-1.06) | 3.60E-01 |
| rs16861329 | 3 | 186666461 | C | T | 1.03 (0.97-1.09) | 3.90E-01 |
| rs6467136 | 7 | 127164958 | A | G | 1.01 (0.98-1.05) | 5.30E-01 |
| rs10886471 | 10 | 121149403 | T | C | 1.01 (0.97-1.05) | 5.90E-01 |
| rs6723108 | 2 | 135479980 | T | G | 1.01 (0.97-1.04) | 7.10E-01 |
| rs9470794 | 6 | 38106844 | T | C | 1.01 (0.95-1.08) | 8.00E-01 |
| rs17584499 | 9 | 8879118 | T | C | 1.00 (0.95-1.06) | 9.40E-01 |
| rs391300 | 17 | 2216258 | C | T | 1.00 (0.96-1.04) | 9.50E-01 |

Shown are 74 autosomal variants associated with T2D in Ref. 10 that were used to construct genetic risk scores in GOMAP. For each variant, chromosome position, effect (EA) and alternative (NEA) allele, as well as odds ratios with 95% confidence interval and p-values are given.

**Supplementary Table 5. Genomic regions with evidence of colocalising signals**

|  |  |  |  |  |  | **DIAGRAMv3** | | | | **PGC-SCZ** | | | |
| --- | --- | --- | --- | --- | --- | --- | --- | --- | --- | --- | --- | --- | --- |
| **chunk** | **SNPs** | **chr** | **start** | **stop** | **PP4** | **max \|Z\|** | **top SNP** | **Closest gene** | **Closest gene dist.** | **max \|Z\|** | **top SNP** | **Closest gene** | **Closest gene dist.** |
| 172 | 1419 | 2 | 60293221 | 62425639 | 0.92 | 5.67532 | rs243019 | BCL11A | 92496 | 4.9996 | rs10189857 | BCL11A | 0 |
| 656 | 160 | 6 | 31704294 | 32634467 | 0.96 | 4.63055 | rs9267576 | C6orf48 | 4497 | 8.88914 | rs3117574 | MSH5,MSH5-SAPCD1 | 0 |
|  |  |  |  |  |  |  | rs9267658 | SLC44A4 | 0 |  |  |  |  |
|  |  |  |  |  |  |  | rs3130285 | TNXB | 0 |  |  |  |  |
| 822 | 2158 | 7 | 130424544 | 132805104 | 1.00 | 6.51052 | rs10954284 | KLF14 | 44870 | 5.60819 | rs7801375 | PLXNA4 | 240828 |
| 854 | 1301 | 8 | 9641034 | 10462806 | 0.92 | 5.30824 | rs17150816 | MSRA | 121041 | 5.26135 | rs11993663 | MSRA | 0 |
| 1405 | 2213 | 15 | 61266132 | 63214206 | 0.99 | 5.64347 | rs8026735 | VPS13C | 26113 | 6.0853 | rs11632947 | RP11-507B12.2 | 0 |
|  |  |  |  |  |  |  | rs875513 | VPS13C | 18729 |  |  |  |  |

Shown are genomic regions (chunks) with a high posterior probability of harbouring two distinct causal variants for T2D and SCZ (PP4>0.9). For each region, the variants with the highest absolute Z score for T2D and SCZ are given, respectively.

**Supplementary Table 6. Variants overlapping at P_t_=0.001 in DIAGRAM and PGC**

|  |  |  |  |  |  |  |  | **DIAGRAM** | | **PGC** | |
| --- | --- | --- | --- | --- | --- | --- | --- | --- | --- | --- | --- |
| **Variant** | **Chr** | **Pos (hg18)** | **EA** | **NEA** | **Gene** | **Distance** | **EAF** | **P** | **OR (95% CI)** | **P** | **OR (95% CI)** |
| rs10792688 | 11 | 83255725 | C | G | *DLG2* | 0 | 0.65 | 9.60E-05 | 0.98 (1.01-0.94) | 5.14E-07 | 1.06 (1.08-1.04) |
| rs11634397 | 15 | 80432222 | A | G | *ZFAND6* | 1487 | 0.66 | 7.30E-06 | 1.07 (1.03-1.11) | 1.47E-04 | 0.96 (1.06-1.02) |
| rs2523589 | 6 | 31327334 | G | T | *HLA-B* | 2369 | 0.53 | 4.20E-05 | 1.10 (1.07-1.14) | 1.00E-01 | 0.98 (1.04-1.00) |
| rs27419 | 5 | 52420938 | C | T | *MOCS2* | 15045 | 0.68 | 1.00E-04 | 1.00 (1.03-0.96) | 1.19E-09 | 1.07 (1.09-1.05) |
| rs275746 | 15 | 40154938 | G | C | *GPR176* | 0 | 0.08 | 7.60E-04 | 1.09 (1.05-1.13) | 8.26E-04 | 1.04 (0.98-0.94) |
| rs2796441 | 9 | 84308948 | G | A | *TLE1* | 4728 | 0.40 | 2.50E-04 | 0.98 (1.02-0.94) | 1.46E-07 | 0.94 (1.08-1.04) |
| rs2838968 | 21 | 47038365 | C | T | *PCBP3* | 25243 | 0.18 | 1.00E-03 | 1.26 (1.13-1.40) | 1.24E-01 | 1.04 (1.01-0.91) |
| rs340835 | 1 | 214163675 | G | A | *PROX1* | 0 | 0.45 | 1.10E-06 | 0.92 (0.95-0.89) | 1.38E-02 | 1.03 (1.05-1.01) |
| rs5757761 | 22 | 40054948 | T | C | *CACNA1I* | 0 | 0.46 | 7.10E-04 | 1.08 (1.04-1.12) | 9.69E-07 | 1.06 (1.08-1.04) |
| rs6488868 | 12 | 123799974 | A/G | T | *SBNO1* | 0 | 0.70 | 5.10E-04 | 0.93 (0.96-0.89) | 9.92E-06 | 0.95 (0.97-0.93) |
| rs6752494 | 2 | 200313323 | T | C | *SATB2* | 0 | 0.17 | 2.10E-04 | 0.87 (0.94-0.81) | 1.64E-04 | 1.09 (0.96-0.88) |
| rs6999153 | 8 | 9193501 | G | A | *RP11-10A14.4* | 167855 | 0.56 | 2.00E-04 | 1.07 (1.03-1.12) | 2.21E-07 | 1.05 (0.97-0.93) |
| rs7450789 | 6 | 111816975 | G | T | *REV3L* | 12057 | 0.86 | 5.00E-04 | 1.07 (1.03-1.12) | 1.67E-05 | 1.06 (0.97-0.91) |
| rs7614727 | 3 | 52295895 | C | T | *WDR82* | 0 | 0.43 | 8.50E-04 | 0.97 (1.03-0.92) | 6.71E-19 | 1.18 (0.89-0.81) |
| rs7652609 | 3 | 1812610 | C | A | *CNTN4* | 327887 | 0.06 | 8.90E-04 | 1.12 (1.06-1.18) | 2.65E-02 | 0.96 (1.07-1.01) |
| rs7682321 | 4 | 19099432 | A | G | *LCORL* | 1075933 | 0.26 | 9.70E-04 | 0.92 (0.95-0.89) | 4.32E-06 | 1.05 (0.97-0.93) |
| rs7783665 | 7 | 110039196 | G | A | *IMMP2L* | 263914 | 0.42 | 4.10E-05 | 0.93 (0.97-0.90) | 2.64E-05 | 0.95 (0.97-0.93) |
| rs8192675 | 3 | 170724883 | T | C | *SLC2A2* | 0 | 0.29 | 7.00E-04 | 0.93 (0.97-0.90) | 1.82E-04 | 0.95 (1.07-1.03) |
| rs830644 | 3 | 71665559 | T | C | *FOXP1* | 32419 | 0.57 | 3.60E-04 | 0.90 (0.95-0.85) | 2.32E-06 | 1.08 (1.11-1.05) |

Shown are the 19 independent variants overlapping at a p-value threshold of 0.001 between summary statistics from DIAGRAM and PGC.

**Supplementary Table 7. Significantly associated genes in DIAGRAM and PGC**

| **Gene** | **DIAGRAM q-value** | **PGC q-value** | **CHR** | **Start_hg19** | **Stop_hg19** | **Strand** |
| --- | --- | --- | --- | --- | --- | --- |
| *HLA-DRB1* | 2.33E-02 | 1.21E-06 | 6 | 32546546 | 32557613 | - |
| *KCNJ11* | 3.99E-02 | 1.56E-02 | 11 | 17406795 | 17410878 | - |
| *TSPAN8* | 2.02E-02 | 2.49E-02 | 12 | 71518877 | 71551779 | - |
| *ZFAND6* | 1.75E-02 | 7.25E-03 | 15 | 80351910 | 80430735 | + |
| *SSR1* | 3.70E-02 | 4.12E-02 | 6 | 7281283 | 7313541 | - |
| *SLC9B2* | 2.04E-02 | 9.32E-04 | 4 | 103946647 | 103998480 | - |
| *CISD2* | 2.18E-02 | 6.61E-04 | 4 | 103749224 | 103813964 | + |
| *UBE2D3* | 2.16E-02 | 6.52E-04 | 4 | 103715540 | 103790050 | - |
| *SLC9B1* | 2.06E-02 | 1.30E-06 | 4 | 103806205 | 103947552 | - |
| *PRRT1* | 2.06E-02 | 8.02E-03 | 6 | 32116140 | 32119720 | - |
| *EHBP1L1* | 2.94E-02 | 2.63E-02 | 11 | 65343509 | 65360121 | + |
| *PROX1* | 2.24E-02 | 9.89E-03 | 1 | 214161278 | 214214853 | + |
| *CDKAL1* | 4.73E-06 | 2.63E-02 | 6 | 20534688 | 21232635 | + |
| *RCCD1* | 2.02E-02 | 4.98E-02 | 15 | 91498106 | 91506355 | + |
| *NFKBIL1* | 2.24E-02 | 2.57E-03 | 6 | 31514628 | 31526606 | + |
| *NEU1* | 2.28E-02 | 8.92E-11 | 6 | 31826829 | 31830709 | - |
| *MICB* | 3.68E-02 | 2.63E-06 | 6 | 31462054 | 31478901 | + |
| *EHMT2* | 2.02E-02 | 4.16E-10 | 6 | 31847536 | 31865464 | - |
| *HLA-B* | 4.67E-02 | 1.69E-10 | 6 | 31321649 | 31324989 | - |
| *ATP6V1G2* | 2.06E-02 | 2.61E-03 | 6 | 31512228 | 31514625 | - |
| *DDX39B* | 2.06E-02 | 6.80E-04 | 6 | 31497996 | 31510252 | - |
| *GIP* | 2.66E-02 | 9.32E-04 | 17 | 47035918 | 47045955 | - |
| *SNF8* | 4.84E-02 | 3.35E-03 | 17 | 47007458 | 47022484 | - |
| *UBE2Z* | 4.88E-02 | 5.51E-03 | 17 | 46985731 | 47006422 | + |
| *ZBTB12* | 8.57E-03 | 2.43E-04 | 6 | 31867394 | 31869769 | - |
| *MCCD1* | 2.94E-02 | 2.37E-05 | 6 | 31496739 | 31498008 | + |
| *SLC44A4* | 2.02E-02 | 4.73E-11 | 6 | 31830969 | 31846823 | - |
| *KCNK7* | 4.18E-02 | 4.32E-03 | 11 | 65360326 | 65363467 | - |
| *UNC45A* | 2.71E-02 | 3.56E-02 | 15 | 91473410 | 91497323 | + |

Shown are 30 genes with significant evidence (q-value<0.05) of association in both DIAGRAM and PGC.
